# Elevated Expression of the *RAGE* Variant-*V* in SCLC Mitigates the Effect of Chemotherapeutic Drugs

**DOI:** 10.3390/cancers13112843

**Published:** 2021-06-07

**Authors:** Bindhu K. Madhavan, Zhe Han, Bishal Singh, Nico Bordt, Serap Kaymak, Obul Reddy Bandapalli, Lars Kihm, Khurrum Shahzad, Berend Isermann, Stephan Herzig, Peter Nawroth, Varun Kumar

**Affiliations:** 1 Department of Medicine I and Clinical Chemistry, University Hospital of Heidelberg, INF 410, 69120 Heidelberg, Germany; bindhu31779@gmail.com (B.K.M.); zhe.han@med.uni-heidelberg.de (Z.H.); bishaliitg2004@gmail.com (B.S.); ni.co.4@web.de (N.B.); serap.kaymak@med.uni-heidelberg.de (S.K.); Lars.kihm@med.uni-heidelberg.de (L.K.); Peter.nawroth@med.uni-heidelberg.de (P.N.); 2 Hopp Children’s Cancer Center (KiTZ), 69120 Heidelberg, Germany; o.bandapalli@kitz-heidelberg.de; 3 Medical Faculty, Heidelberg University, 69117 Heidelberg, Germany; 4 Institute for Laboratory Medicine, Clinical Chemistry and Molecular Diagnostics, University Hospital Leipzig, 04103 Leipzig, Germany; khurrum.shahzad@medizin.uni-leipzig.de (K.S.); berend.isermann@medizin.uni-leipzig.de (B.I.); 5 German Center for Diabetes Research (DZD), 85764 Neuherberg, Germany; stephan.herzig@helmholtz-muenchen.de; 6 Molecular Metabolic Control, Technical University Munich, 80333 Munich, Germany; 7 Helmholtz Center Munich, Institute for Diabetes and Cancer, D-85764 Neuherberg, Germany; 8 Joint Heidelberg-IDC Translational Diabetes Programm, Helmholtz-Zentrum, 69120 Heidelberg, Germany; 9 European Molecular Biology Laboratory, Advanced Light Microscopy Facility, 69117 Heidelberg, Germany

**Keywords:** DNA repair, small cell lung carcinoma, RAGE, laser induced DNA damage, migration, invasion, wound healing

## Abstract

**Simple Summary:**

Radiomimetic drugs induce extensive genotoxic insults to their target cells. Irreparable DNA damage leaves cells with the choice between a program leading to cell death or senescence, but not DNA repair. Among the challenges of an advanced stage of small cell lung carcinoma (SCLC), the resistance to radiomimetic drugs is the most prominent one. In SCLC, the initial chemotherapeutic treatment primes cell to modify their DNA repair and cell cycle regulatory systems, using alternative but highly efficient forms of DNA repair and auxiliary factors. This modulated system now bypasses several regulatory controls. Thus, at this stage, cells become resistant to any beneficial effects of chemotherapeutic drugs. In the present study, we observed that variant-V of the receptor for advanced glycation end-products (RAGE) is abundantly expressed in advancing and metastasizing SCLC. Therefore, it may serve as a potential target for specific therapeutic interventions directed to SCLC.

**Abstract:**

Small cell lung carcinoma (SCLC) is a highly aggressive malignancy with a very high mortality rate. A prominent part of this is because these carcinomas are refractory to chemotherapies, such as etoposide or cisplatin, making effective treatment almost impossible. Here, we report that elevated expression of the *RAGE* variant-*V* in SCLC promotes homology-directed DNA DSBs repair when challenged with anti-cancer drugs. This variant exclusively localizes to the nucleus, interacts with members of the double-strand break (DSB) repair machinery and thus promotes the recruitment of DSBs repair factors at the site of damage. Increased expression of this variant thus, promotes timely DNA repair. Congruently, the tumor cells expressing high levels of variant-*V* can tolerate chemotherapeutic drug treatment better than the RAGE depleted cells. Our findings reveal a yet undisclosed role of the *RAGE* variant-*V* in the homology-directed DNA repair. This variant thus can be a potential target to be considered for future therapeutic approaches in advanced SSLC.

## 1. Introduction

Small cell lung carcinoma (SCLC) represents one of the most aggressive and lethal forms of lung cancer [1]. It accounts for about 16% of the total lung cancer cases worldwide and is prominently linked to mutagens associated with tobacco smoke [2]. At the morphology level, SCLC is characterized by intense tumor cell mass growth, vascularization, deregulation of cell cycle checkpoints and signaling networks [3,4,5]. Furthermore, timely SCLC diagnosis and combinatorial treatment with radiomimetic drugs (such as cisplatin and etoposide) improve the short-term survival rate. Still, acquired resistance to chemotherapeutics with very early relapses is frequently causing treatment failures. Most importantly, SCLC also presents additional challenges to the clinician such as frequent paraneoplastic endocrinopathies and metastasis [6]. Therefore, the average five years survival rate is only 6%.

More importantly, the early post combinatorial therapy resistance in SCLC is linked to a highly active and efficient DNA repair system [1,7]. Thus, it is very difficult to challenge them with alternative anti-cancer drugs that commonly target genomic integrity [1,8]. In a normal cell, the DNA damage response (DDR) involves the timely and concerted action of various repair factors which sense, signal and repair the DNA damage by either an error-free homology-directed repair (HDR) or an error-prone non-homologous end-joining (NHEJ) repair [9]. Thus, on-demand activation of the DNA repair pathway plays an essential role in preserving the genetic integrity of a healthy cell [10]. Conversely, the SCLC cells exploit this crucial cellular process for their survival when challenged with genotoxic agents. To overcome these therapeutic limitations, recent efforts are directed towards developing specific therapeutics for resistant SCLC by focusing on DDR and repair factors, which are abundantly expressed in these resistant cancer cells.

In the lung, the pattern recognition receptor RAGE is constitutively expressed and localizes to the cell surface, mitochondria and nucleus [11,12]. Recently it has been observed that RAGE in the nucleus interacts with the MRN (Mre11-Rad50-Nbs1) complex and participates in the processing of the broken ends of the DNA. Nuclear RAGE thus plays an important role in maintaining genomic integrity [11,13,14]. The RAGE^−/−^ mice have more incidences of lung carcinomas [11]. In addition, to its nuclear function, RAGE, at the cell surface, interacts with AGEs, S100-proteins, amyloid-β-peptides to modulate various signaling cascades [15]. In mitochondria, RAGE participates in energy homeostasis [12]. All vertebrates are known to express various RAGE splice variants of RAGE in addition to RAGE^WT^ [16,17,18,19,20,21]. These alternative splice variants of RAGE might be playing an essential role in expanding the functional diversity of this cellular factor by changing the primary amino acid sequence and potentially also its function. Since the first identification of these variants, the functional role of either of these variants remained obscure.

In the present study, we identified and characterized a novel variant of RAGE, variant-V (vRAGE). At the organization level, this variant lacks both the transmembrane and the cytoplasmic tail. Moreover, both these structural units were replaced by a robust nuclear localization signal; therefore, *v*RAGE constitutively localizes to the nucleus only and participates in the DNA repair process. Contrary to the RAGE^WT^ expression in lung cancer, the variant RAGE is abundantly expressed in SCLC biopsies and SCLC derived cells. Therefore, vRAGE might play an important role in chemoresistance. Thus, targeting this vRAGE and the NHEJ repair pathway, in pre- or resistant stages of SCLC might be a future option for developing SCLC therapies specific for resistant cancer cells.

## 2. Results

### 2.1. Both RAGE^WT^ and the Variant RAGE-V Share an Identical Structural Organization of the DNA Binding Modules of RAGE

**Figure 1:**
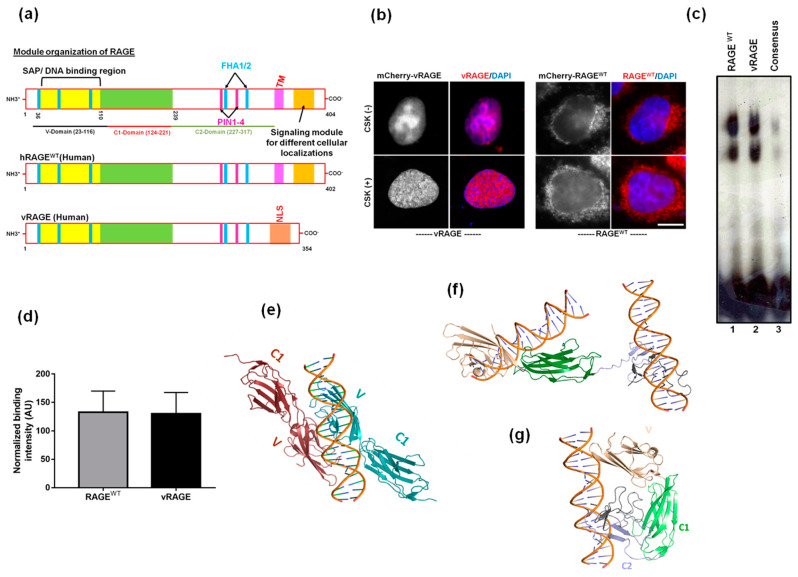
Comparative analysis of DNA binding abilities of RAGE^WT^ and vRAGE. (**a**) A comparative pictorial representation of domains associated with the nuclear function of RAGE^WT^ and a RAGE variant-V (vRAGE). The positions of domains (V, C1 and C2) are marked below the respective representation. (**b**) Representative images of Hela cells ectopically expressing mCherry-vRAGE (left) or mCherry-RAGE^WT^ (right) shows the localization of vRAGE or RAGE^WT^ (red) and the DAPI (Blue). These cells were either left un-extracted (−) or were extracted (+) with CSK buffer before fixation. The fluorescence of mCherry (no antibody) was used for detecting the vRAGE or RAGE^WT^. Blue color represents DAPI (Scale 6 µm). (**c**) In a native gel shift assay, a 64mer dsDNA (~60ng), labeled at the 5′ end of one of the strands and was incubated with either RAGE^WT^ or vRAGE (25nM) as described in methods. The consensus served as a negative control. (**d**) Densitometry quantification plot of the gel shift assay described in figure (Figure 1c). (*n* = 2) (**e**) The ribbon-diagram illustration of the nucleic acids bound human RAGE^WT^ crystal structure (PDB accession code 4oi7). Nucleic acids interact largely at the dimeric interface between variable domains (V). The C2 domain is not present in the crystal structure. (**f**) Ribbon-diagram illustration of a nucleic acid sequence containing the vRAGE model structure after molecular docking. This shows that the c-terminal region (C2) of vRAGE is also capable of binding to the DNA. (**g**) The ribbon-diagram illustration of a nucleic acid sequence with the v RAGE model structure in a bent state after molecular docking and structural simulation.

RAGE and its splice variants are abundantly expressed in the lungs of all vertebrates [22]. To determine the functional importance of these splice variants, comparative sequence-based in silico analysis of both human RAGE^WT^ and RAGE variant-V (hereafter, we refer to it as vRAGE) was performed. It was observed that vRAGE retains the N-terminus of RAGE^WT^, including the DNA binding region and the predicted epigenetic regulated regions (Figure S1a). The c-terminus of RAGE^WT^ plays an essential role in regulating the various cellular localizations of RAGE, on the cell surface, mitochondria and the signal-dependent nuclear localization [11,12,23]. The c-terminus, however, differs significantly between these two isoforms of RAGE. More specifically, vRAGE lacks a membrane-spanning domain and the cytoplasmic tail and these regions were replaced with a robust nuclear localization signal (NLS), indicating that this variant of RAGE might be a constitutive nuclear factor (Figure 1a). To compare the importance the predicted NLS at the c-terminus with the c-terminus of classical RAGE (RAGE^WT^), a clone expressing either mCherry tagged vRAGE, or RAGE^WT^ was transfected into Hela cells. After about 24 h of transfection, RAGE localization was determined by the indigenous mCherry fluorescence. It was observed that vRAGE constitutively localizes to the nucleus, whereas the RAGE^WT^ prominently resides in the cytosol of the resting Hela cells (Figure 1b). Thus, the c-terminal NLS serves as a potent nuclear localization signal.

Moreover, to test if vRAGE in the nucleus also interacts with DNA, transfected cells were pre-extracted with CSK buffer [24]. The fluorescence analysis shows that vRAGE indeed interacts with chromatin (Figure 1b). To further validate whether the interaction of vRAGE with chromatin is direct, or mediated through other factor(s), vRAGE and RAGE^WT^ proteins were expressed and purified from an *E. coli* expression host [11,25]. These purified factors were then used in an in vitro gel retardation assay, by using a radiolabeled probe, as described earlier [11]. The EMSA result shows that dsDNA interactions with both RAGE^WT^ and vRAGE are comparable (Figure 1c,d). Moreover, to evaluate the contribution of the c-terminal region in DNA-dependent functions of RAGE, a sequence-based homology modeling and the molecular docking of dsDNA on both RAGE^WT^ and vRAGE was performed (Figure S1b). The structural topology in the modeled structure of vRAGE is identical to the crystal structure of the RAGE^WT^ at V- and C1-domain, but it offers a distinct pattern at the c-terminus (Figure S1b). The c-terminus of RAGE^WT^ plays an essential role in the dimerization of RAGE^WT^ without any significant contribution to DNA binding directly [26]. Striking structural differences at the c-terminal of vRAGE model structure hints at the existence of vRAGE monomers in the physiological state. Molecular docking of dsDNA with the vRAGE model structure revealed that in contrast to the RAGE^WT^, where dsDNA binds with the V domain at the dimer interface (Figure 1e), both the V-domain and the C-terminus domain of vRAGE are able to interact with dsDNA (Figure 1f). Further structural refinement and simulation of dsDNA bound vRAGE model structure revealed that the c-terminus in vRAGE can fold itself toward the V domain, affirming dsDNA binding (Figure 1g). This indicates that vRAGE is able to interact with DNA as a monomer, whereas dimerization of RAGE^WT^ is essential for interacting with dsDNA.

### 2.2. vRAGE is Involved in the DSB Repair as an Early DDR Factor

**Figure 2:**
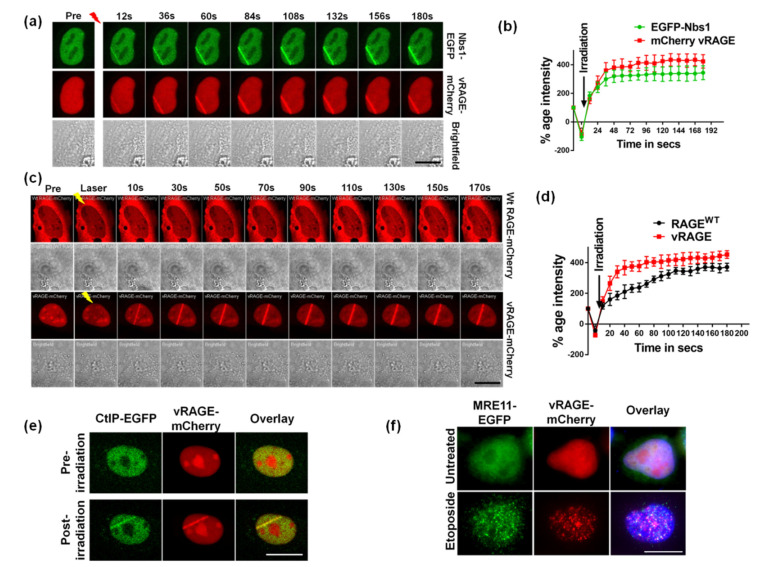
DNA damage-induced recruitment of vRAGE at the site of DSBs. (**a**) The representative still images of Hela cells show the live recruitment of human vRAGE-mCherry and human Nbs1-EGFP at the site of laser-induced DNA-DSBs at the indicated time interval. The brightfield images are presented in gray (Scale 10 µM). (**b**) Quantitative plot showing the normalized intensity of the EGFP tagged-NBS1 (green line) or mCherry-tagged vRAGE (red line) at the site of damage (*n* = 5). (**c**) The representative still images of comparative live recruitment kinetics of RAGE^WT^-mCherry and vRAGE-mCherry at the indicated time intervals in Hela cells ectopically expressing the indicated factors (Scale 10 µM). (**d**) Quantitative plot showing the normalized intensity of the mCherry-RAGE^WT^ (black line) or mCherry-vRAGE (red line) at the site of damage (*n* = 5). (**e**) The representative still images of Hela cells expressing hCtIP-EGFP and vRAGE-mCherry from the live recruitment studies show the co-localization of vRAGE and CtIP at the site of laser-induced DNA damage. The pre-irradiation image represents the pre-laser induced DNA damage control. The images presented show the native fluorescence of each fluorochrome (Scale 10 µM). (**f**) Representative immunofluorescence images showing the co-localization of MRE11 and vRAGE in Hela cells expressing hMRE11-EGFP and vRAGE-mCherry, either left untreated, or treated with etoposide (2.5 μM for 1hour), analyzed for co-localization of MRE11 (green) with vRAGE (red). DAPI (blue) served as a nuclear stain. These cells were detergent extracted before fixing in 4% Paraformaldehyde (Scale 10 µM).

RAGE plays a vital role in DNA double-strand break (DSBs) repair [11,14]. The vRAGE is constitutively localized to the nucleus. Thus, to understand the role of vRAGE in the nucleus, particularly in DNA repair, we used Hela cells ectopically expressing EGFP-tagged Nbs1 (EGFP-Nbs1; human), a positive control for the recruitment of the DNA repair machinery and mCherry-tagged vRAGE (human) as the factor to study. These transfected cells were then used for laser micro-irradiation mediated DNA damage studies by tracking the live recruitment kinetics of these transfected factors at the site of DNA damage [14]. To prevent the non-specific bleeding of GFP or mCherry fluorescence into each other, a sequential mode of live imaging was used. As expected, induction of DNA DSB leads to the immediate recruitment of EGFP-Nbs1, usually within 12 s post DNA damage showing that DSB repair pathways were activated by microirradiation of these cells (Figure 2a,b). Interestingly, in addition to EGFP-Nbs1, the mCherry-tagged vRAGE was recruited to the site of the damage already within 12 s. This indicates that vRAGE serves as a downstream target DNA damage response (Figure 2a,b). The DNA damage-dependent recruitment of vRAGE to the site of damage was also validated in U2OS (Figure S2) and A549 cells (Figure S2).

Both RAGE^WT^ and vRAGE recruit to the site of damage. In order to understand the molecular differences between these two forms of human RAGE (RAGE^WT^ vs. vRAGE), live microscopy studies were performed by using either mCherry-tagged RAGE^WT^ or vRAGE expressing Hela cells. The live recruitment kinetic data shows that vRAGE recruitment was faster and more intense than that of RAGE^WT^ when studied under the same conditions (Figure 2c,d). The RAGE^WT^ recruits to the site of damage in around ~50 s, whereas the vRAGE comes to the site of damage in less than 10 s (Figure 2c,d). This shows that kinetically vRAGE is about 5 times faster at the site of damage than RAGE^WT^. As both RAGE^WT^ and vRAGE retained the ability to interact with dsDNA and retain all the DNA interaction modules, the observed difference can be explained by the additional time required for translocation of RAGE^WT^ to the nucleus (and to the site of damage). In contrast, the constitutive nuclear presence seems advantageous to vRAGE (Figure 2c,d).

To further validate the involvement of vRAGE in DNA DSB repair, the DNA damage-induced co-localization of vRAGE with other DNA repair markers was tested. Two different approaches were used. In the first approach, the EGFP-tagged CtIP (CtIP-EGFP) and mCherry tagged vRAGE (vRAGE-mCherry) expressing cells were used for laser microirradiation and the co-localization analysis was performed [27]. The co-localization result shows that both CtIP-EGFP and vRAGE-mCherry co-localize at the site of DNA damage (Figure 2e). Further to quantitatively evaluate the co-localization, the Pearson R-coefficient (R) was calculated [28]. The co-localization coefficient for CtIP-EGFP with vRAGE-mCherry was 0.21 before irradiation and increased to 0.94 after irradiation, indicating the DNA damage mediated CtIP/vRAGE co-localization.

In the second approach, etoposide-mediated DNA damage was induced in Hela cells expressing MRE11-EGFP as well as vRAGE-mCherry and the native fluorescence of the indicated tags was used for co-localization analysis. Similar to laser irradiation, etoposide enhances the co-localization of MRE11 to vRAGE (R = 0.74), whereas the untreated cells show prominent co-localization (R = 0.28) (Figure 2e). Thus, both these studies show that vRAGE co-localizes with the DNA DSB marker CtIP (Figure 2e) and the MRE11 foci (Figure 2f). Thus, vRAGE is an early DNA damage repair factor that stimulates DNA repair in a kinetically much more efficient way than its RAGE^WT^ counterpart. Moreover, at the site of damage, it co-localizes with the DSB repair machinery.

### 2.3. vRAGE Stimulates the DNA DSBs Repair and Thus, Enhances the Repair Potential of Cells

**Figure 3:**
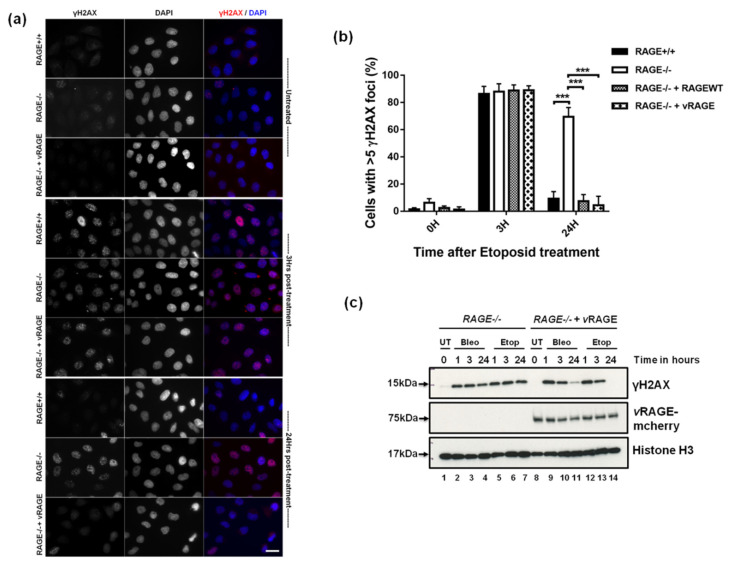
DNA damage-induced recruitment of vRAGE at the site of DSBs. (**a**) Immunofluorescence analysis of DSB associated foci as marked by γH2AX in un- or vRAGE complemented Hela^−/−^ cells, treated with etoposide (2.5 µM for 1 h). Wild-type Hela cells (Hela^+/+^) served as an experimental control. The resolution of DNA-DSB foci, as marked by γH2AX, was monitored over 3 and 24 h after drug treatment; DAPI represents the nucleus (scale 10 µm). (**b**) Mean percentage of DSBs marker γH2AX positive nuclei of Hela cells as described in Figure 3a and Figure S4a. More than 400 cells were analyzed for each bar (mean ± SD, ***: *p* <; 0.001). (**c**) Representative immunoblots from the lysates of un-complemented (RAGE^−/−^) or vRAGE complemented (RAGE^−/−^ + vRAGE) RAGE^−/−^ Hela cells, treated with etoposide (2.5 μM for 1 h) or bleomycin (30 µg/mL for 1 h) and probed for the DNA damage marker γH2AX at the indicated intervals after DNA damage. The expression of vRAGE was confirmed by using a vRAGE specific antibody. Histone H3 is used as a loading control.

To understand the importance of vRAGE in the DNA repair process, CRISPR-Cas9 generated RAGE^−/−^ Hela cells were used (Figure S3a,b). Usually, Hela and other eukaryotic cells can repair the DNA damage induced by radiation or chemotherapeutic drugs within 24 h, provided they possess a functional DNA repair system [27]. Thus, etoposide (2.5 µM for 1 h) mediated DNA DSBs were induced in Hel^−/−^ cells that were either left uncomplemented, or were complemented with vRAGE-mCherry expressing clones. Hela^WT^ (Hela^+/+^) and Hela^−/−^ cells complemented with RAGE^WT^ served as an internal control. For the comparative DNA repair analysis, the DSBs signaling marker γH2AX was used [14,29,30]. Upon etoposide treatment, all group cells (Hela^−/−^, Hela^−/−^, Hela^−/−^ + RAGE^WT^ and Hela^−/−^ + vRAGE) show intense γH2AX positivity, as determined by immunofluorescence staining (Figure 3a and Figure S4a). This indicates that neither the absence of RAGE, nor the over-expression of RAGE (vRAGE or RAGE^WT^) interferes with the DNA damage sensing and signaling. When the DNA repair kinetics of these groups were followed over a complete repair cycle (i.e., up to 24 h post-removal of drug), the RAGE^−/−^ cells could not repair the DSBs within 24 h. This indicates that the absence of RAGE compromises the DNA repair system. More importantly, RAGE^−/−^ cells, when complemented with vRAGE-mCherry could repair their DNA DSBs efficiently, showing that vRAGE could complement the defective DNA repair system of RAGE^−/−^ cells to an efficient one (Figure 3a). In addition, complementation of Hela^−/−^ cells with RAGE^WT^ also timely turned-off the DSBs signaling (Figure 3a and Figure S4a). Additionally, the absence of RAGE leads to the accumulation of few γH2AX foci from the DNA damage that might have generated by endogenous metabolic stress or from replication errors (Figure 3a). These data indicate that RAGE (vRAGE or RAGE^WT^) plays an important role in DNA repair. Moreover, to represent the global view of the DNA repair process in these indicated groups, the γH2AX signal was quantified at both the immunofluorescence (Figure 3b) and immunoblotting level (Figure S4b).

To prove that this phenotype is not just related to an etoposide specific effect, another drug, bleomycin (30 µg/mL for 60 min), was used for inducing the DNA damage. The DNA repair kinetics were studied in a similar way as done for etoposide by using the DNA damage marker γH2AX. In this assay, etoposide treatment served as an internal positive control. Identical to the previous observation with etoposide, induction and follow-up repair analysis showed that Hela^−/−^ cells complemented with vRAGE, can repair their DSBs timely. This repair process is showing similar kinetics for both bleomycin and etoposide (Figure 3c), whereas the absence of RAGE (vRAGE or RAGE^WT^) affects the integrity of the repair system, as marked by the presence of a γH2AX signal after 24 h of induction of DNA damage (Figure 3c). These analyses show the drug independent but DSBs dependent effects of vRAGE in DNA repair.

Moreover, to validate the observation in a cell-type independent manner, RAGE^−/−^ primary fibroblasts isolated from RAGE^−/−^ mice were used [31]. These isolated cells were then either left un-complemented or were complemented with vRAGE. The complementation of RAGE^−/−^ lung fibroblasts with RAGE^WT^ served as an internal control of the experiment. Post transfection (36 h), the DNA repair potential of these cells was studied similarly as described for Hela^−/−^ cells. Similar to the observation made in Hela cells, complementation of vRAGE to RAGE^−/−^ fibroblasts enhances their DNA DSB repair abilities such that these cells can repair the etoposide-induced DNA damage within 24 h, as shown by the DNA damage marker γH2AX (Figure S4c), whereas uncomplemented cells show active DNA damage signaling even after 24 h (Figure S4c). RAGE^−/−^ fibroblasts complemented with RAGE^WT^ serve as positive control of this reconstitution experiment also showed similar repair kinetics as observed in vRAGE complementation (Figure S4c).

### 2.4. vRAGE Is Linked to Aggressively Metastasizing Small Cell Lung Carcinoma

Previously the expression of RAGE has been shown to vary across various cancers. In both non-small cell lung carcinoma (NSCLC) and small cell lung carcinoma (SCLC), the expression of the soluble form of RAGE (known as sRAGE) decreases with the progression of cancer [32,33,34,35]. Since there are various isoforms of RAGE in both humans and mice [19], there is a need to evaluate the importance of increased DNA repair potential linked to the isoform vRAGE in these diseases. Thus, we first generated a specific monoclonal antibody that exclusively recognizes vRAGE, but not RAGE^WT^ (Figure S5a).

Thus, to validate the importance of vRAGE in cancer, cancer samples collected after surgical resection was obtained from Pantomics Inc. (MTU951; Fairfield, CA, USA; https://www.biomax.us/tissue-arrays/Multiple_Organ/MTU951 (accessed on 19 April 2021)) and were stained using the vRAGE specific antibody. From this preliminary screening, it was observed that metastasizing SCLC and many others showed increased expression of vRAGE This supported our recent work, showing that the absence of RAGE increases the lung cancer incidence in the RAGE^−/−^ mice model [11,36]. This indicates that the expression of vRAGE might be linked to the biology of human SCLC. Moreover, to expand the horizons of this observation, SCLC samples were now only focused on. As SCLC is linked to high proliferation, metastasizing abilities and high rates of radiomimetic drug resistance, SCLC cancer samples were obtained from three different stages of this cancer. In total, about 80 SCLC samples (early-stage (22 patients), mid-stage (40 patients), late-stage (18 patients)), along with 20 healthy control lungs, were simultaneously studied and analyzed. To keep an independent characterization of the cancer stage material, cancer and control material were obtained from Pantomics inc. USA (LC10010d, https://www.biomax.us/tissue-arrays/Lung/LC10010d (accessed on 19 April 2021)). The anthropometric details of these patients are given in the table and figure (Table 1, Figure S5b).

**Figure 4:**
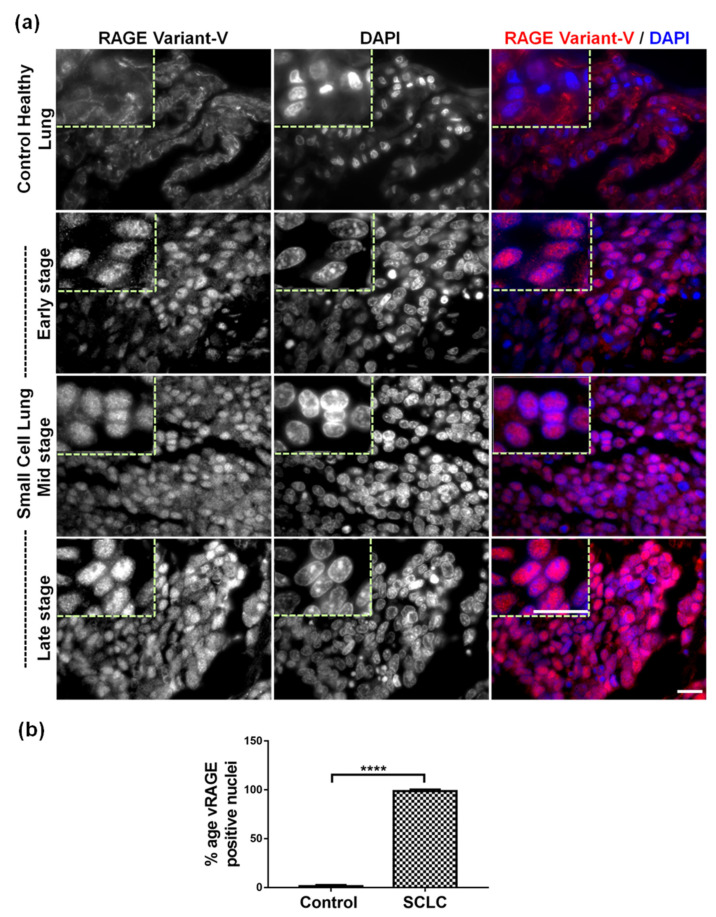
Expression of vRAGE in metastasizing human SCLC samples. (**a**) The representative immunofluorescence images of human lungs diagnosed at different stages of small cell lung carcinoma progression or the healthy control lungs. Staining was performed using a vRAGE specific antibody. The red-stained areas represent the vRAGE specific signal. The DAPI (in blue) represents the nucleus (Scale 10 µm). Anthropometric details of these patients are given in Table 1. (**b**) The quantitative analysis showing the percentage nuclear positivity of the indicated sections for vRAGE antibody. (n^SCLC^ = 80 and n^Control^ = 20, Data presented as mean ± SD, ****: *p* ≤ 0.0001).

However, before studying the vRAGE expression in these samples, the histological examination of these samples was performed, showing different stages of progression of SCLC, whereas the control lung shows a healthy morphology (Figure S5c). Immunofluorescence staining analyses of these samples show that these metastasizing SCLCs samples show very high positivity to vRAGE expression. In contrast, the control lung shows a very low or just detectable expression of this variant of RAGE (vRAGE) (Figure 4b). More importantly, the vRAGE signal intensity increased with the stage of SCLC progression (Figure 4b). This indicates that the elevated expression of this variant of RAGE (vRAGE) in human SCLC might be linked to progression.

Further, many antibodies cross-react with other non-specific epitopes in immunofluorescence staining; thus, to rule out the possibility of non-specific detection, the vRAGE antibody was competed with vRAGE specific or a non-specific peptide during SCLC staining. The vRAGE staining could only be competed (nuclear and foci staining) using the vRAGE specific peptide, but not by non-specific peptide (Figure S5d).

**Figure 5:**
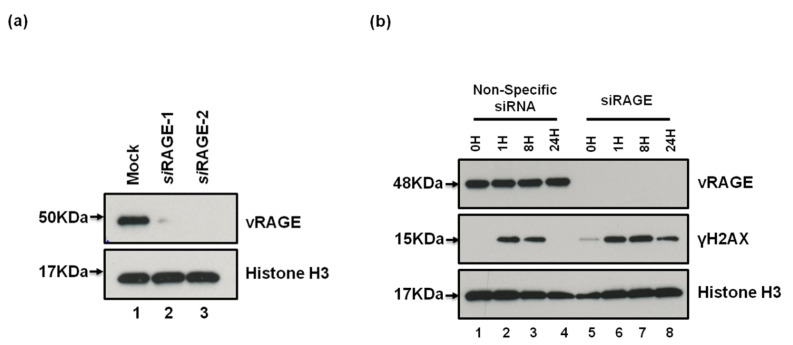
Importance of vRAGE in timely DNA damage response of SCLC cells. (**a**) Representative immunoblots showing the siRAGE (siRAGE1 and siRAGE2) mediated depletion of vRAGE from SHP-77 cells after 72 h of transfection. The mock transfection served as an internal control. (**b**) Representative immunoblots from the lysates of control or RAGE depleted SHP-77 cells (siRAGE-2), treated with etoposide (2.5 μM for 1 h) and probed for the DNA damage marker γH2AX at indicated post-damage intervals. The expression/depletion of vRAGE was confirmed by using a vRAGE specific antibody generated during this study. Histone H3 used as a loading control. The images of uncropped western blots are shown in Figure S8.

To directly evaluate the importance of vRAGE expression in this cancer model, two different SCLC-specific cell lines (SHP77 and NCI H82) were used. The expression of vRAGE in these cells was confirmed as well as validated by using two different human RAGE-specific siRNA-1 and 2 (Figure 5a). Since the siRAGE-2 showed better efficiency, therefore, it was used in DSBs repair kinetic studies. Moreover, all the available SCLC specific cell lines, including SHP77 and NCI H82, grow as suspension culture; therefore, neither colony formation assay, nor live microscopy studies were possible. Therefore, the DNA repair kinetics was studied by immunoblotting using the DSB marker γH2AX.

Depletion of vRAGE from SHP77 cells slows the DNA repair potential of these cells, compared to controls of post-etoposide-induced DSBs (Figure 5b). Moreover, both control and vRAGE depleted cells show comparative γH2AX levels at 1 h post damage, which gets resolved over time in WT SHP77 cells, but not in vRAGE depleted SHP77 cells (Figure 5b). Similar to SHP77 cells, NCI H82 cells (another SCLC-specific cell line) also showed similar DNA repair kinetics (Figure S6), thus validating the importance of vRAGE in the timely DNA repair response of these cells.

Taken together, these data indicate that over-expression of vRAGE; along with other DNA repair factors in SCLC might functionally protect these cells against genotoxic insults. Therefore, these cancers probably show a very high DNA repair rate and causatively attain resistance to radiomimetic drug-induced cell death. Thus, the over-expression of vRAGE along with other DNA repair factors protects these cells from toxic effects of chemotherapeutics.

### 2.5. Radiomimetic Drug Resistance and Metastasis are Cumulatively Associated with vRAGE Over-Expression

**Figure 6:**
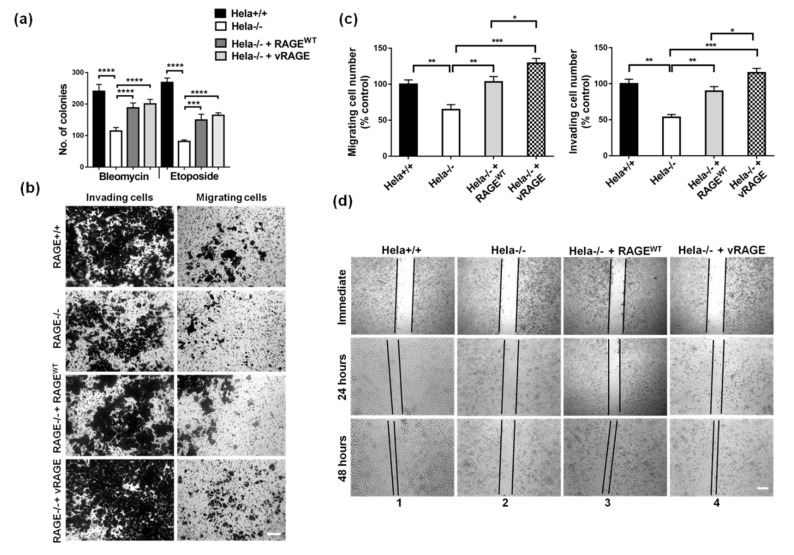
vRAGE is associated with radiomimetic drug resistance, migration, invasion and wound healing. (**a**) Quantitative presentation of the total number of colonies from Hela^+/+^, Hela^−/−^, Hela^−/−^ + RAGE^WT^, or Hela^−/−^ + vRAGE cells surviving after bleomycin (30 µg/mL for 1 h) or etoposide (2.5 µM for 1 h) induced DNA damage. The colonies were attained after 10 days of DNA damage recovery. (*n* = 6, mean ± SD, ***: *p* < 0.001, ****: *p* ≤ 0.0001). (**b**) Representative images of the Hela cells migrated or invaded in the transwell migration assay. The migrated cells were stained with Crystal violet (Scale 40 µm). (**c**) The quantitative analysis of the percentage (%) of the indicated Hela cells migrated (left) or invaded (right) in the transwell assay described in figure (Figure 6b). The migrated cells were stained with crystal violet and counted manually. (*n* = 3, mean ± SD, ns: non-significant, *: *p* < 0.05; **: *p* < 0.01; ***: *p* < 0.001). (**d**) The representative images of Hela cell migration in the wound healing assay directly after wounding, at 24 or 48 h after the induction of a wound (Scale 40 µm).

vRAGE expression along with other repair factors, modulates the DNA repair potential of the cells studied. To further understand the adaptive importance of elevated vRAGE expression in SCLC, the prolonged survival abilities of Hela^−/−^ cells ectopically expressing vRAGE compared to controls, were studied after cells were challenged with chemotherapeutic drugs, using the colony-forming assay. Hela^−/−^ cells complemented with RAGE^WT^ serve as an internal control of the experiment. It was observed that cells over-expressing vRAGE tolerate and timely repair the DNA damage induced by bleomycin (Figure 6a and Figure S7a) or etoposide (Figure 4a and Figure S7a). This indicates a better capacity of RAGE expressing cells to survive and the absence of RAGE (vRAGE or RAGE^WT^) compromised the DNA repair potential of these cells. Furthermore, in addition, to the better ability to survive, vRAGE over-expressing Hela^−/−^ cells were also showing an increased formation of holo- and para-clones, indicating that these cells have a high metastatic potential [37,38] (Figure S7b). This supports our notion, that these varying colony morphologies are indeed associated with cancer metastasis.

To validate the importance of vRAGE in metastasis, the effects of vRAGE expression on cell migration was examined by two independent approaches. First, the transwell migration/invasion assay was performed. The absence of RAGE or vRAGE significantly inhibited Hela cell invasion (Figure 6b,c) and cell migration (Figure 6b,c) compared to the controls. More importantly, a comparative analysis of complementation of Hela^−/−^ cells with vRAGE or RAGE^WT^, show that vRAGE significantly enhances both invasion and migration abilities of these cells (Figure 6b,c) in vitro, indicating the importance of vRAGE expression in cancer progression and metastasis. These observations were also validated in SHP77 cells (Figure S7c,d), this validates the importance of vRAGE in the progression and metastasis of SCLC cells. In the second approach, an in vitro wound-healing assay was used for comparing the wound closure rate between controls and vRAGE over-expressing Hela^−/−^ cells. The Hela^−/−^ cells’ wound closure rate was decreased compared to Hela^+/+^ controls after 48 h of healing (Figure 6d and Figure S7e). Furthermore, as observed using the cell invasion assay, over-expression of vRAGE in these Hela^−/−^ cells timely seal the scratched wound (Figure 6d and Figure S7e), supporting the metastatic potential of these cells.

Our data presented collectively shows that expression of vRAGE modulates the DNA repair system of the cell, thus providing a vital, but clinically undesired protections and, finally, resistance of cancer cells against the genotoxic insults. Therefore, specific targeting of vRAGE alone or in combination with others could become a practical approach to combat against chemoresistance, as frequently observed in these patients.

## 3. Discussion

Alternative precursor RNA splicing may alter the native function and/or localization of proteins [35,39]. This study shows that alternative splicing of a RAGE transcript leads to a variant called vRAGE. This variant is characterized by a strong nuclear localization signal (NLS). Its constitutive nuclear localization and timely DNA repair response, compared to RAGE^WT^, cumulatively promotes better DNA repair, chemoresistance, migration and invasion. Future studies are needed to show that this is related to metastasis and altogether poor prognosis in patients whose tumor expresses this variant of RAGE. With the help of a novel antibody, this variant can be detected in tissue as a key marker of radiomimetic drug-resistant SCLC. Furthermore, prospective studies are required to prove that induction of this splice variant marks the point at which chemoresistance and rapid metastasis limit the patient prognosis. The hypothesis that overcoming HR and NHEJ mediated chemoresistance will in the future add to the therapeutic arsenal in SCLC and potentially other types of cancer.

The mechanism shown here is related to constitutive nuclear localization of vRAGE leading to interaction with the MRN complex at the site of damage [11] and is thus distinct from the other established roles of RAGE on the cell surface or in mitochondria [12]. All of the human RAGE variants identified before retaining the extracellular region of the RAGE and alternative splicing generates variation at the c-terminus [18,19,20,21,40]. The c-terminal of RAGE plays an essential role in signaling cascades on the surface of a cell. Thus, the absence of this region in vRAGE indicates alternative roles of RAGE in cellular physiology [41]. Previously RAGE^WT^ has been studied with respect to its role as a transmembrane pattern recognition receptor, which can induce a sustained pro-inflammatory cellular response [42,43,44,45]. However, variant-V lacks the c-terminal transmembrane and tail region. Both of these regions were replaced by a strong nuclear localization signal, explaining the selective nuclear function of vRAGE. Positively charged amino acids at the c-terminal region of vRAGE may also stabilize the vRAGE bound to DNA. Structural modeling of vRAGE followed by its molecular docking with dsDNA also showed that the c-terminus of vRAGE potentially has the ability to interact with dsDNA. These results suggest that the interaction of DNA with vRAGE is probably energetically favored compared to the RAGE^WT^ DNA interaction. There is also a strong probability that in contrast to the RAGE^WT^, the dimerization of vRAGE is not required for binding to DNA. The change in binding and other functional properties of the factors generated through alternative splicing is also common in cell physiology [46,47]. One such example is the estrogen receptor [48]. Similarly, the CHK2 kinase splice variants are known to modulate the kinase activity of CHK2^WT^ kinase, leading to a dominant-negative effect [49]. Thus, the enhanced DNA repair function linked to vRAGE is the overall effect of structural modification and the localization of vRAGE in the nucleus.

At the site of DNA damage, RAGE modulates the broken DNA end-processing step of the DNA repair [11]. Surprisingly, expression of vRAGE not only promotes the survival potential of the cells when challenged with chemotherapeutic drugs, but also cellular properties related to rapid proliferation, adhesion, at the end metastasis, as shown by the in vitro experiments studying cell migration and invasion. This might be explained by the rapid salvation of DNA damage, but it cannot be excluded, that the constitutive nuclear presence of vRAGE redirects gene expression too.

This leads to the yet unsolved question of the regulation of the balance between RAGE^WT^ and vRAGE. When both isoforms of RAGE bind to DNA with similar affinities and function in the DNA repair process, the selective expression of one isoform over the other might have selective advantages. Importantly, the expression of RAGE^WT^ decreases in progressing SCLC [50], but the DNA repair continues with vRAGE. The expression of vRAGE is low in the normal healthy lung, but elevated in SCLC. How the alternative splicing of RAGE is regulated, which signals determine the expression of each variant, whether suppression of vRAGE or induction of vRAGE transcription is driving the progression of SCLC in in vivo remains to be studied. Furthermore, future studies have to show whether the signaling events, that take place during DNA damage and cell cycle arrest, are the same signals which determine the balance between RAGE^WT^ and vRAGE.

SCLC and other metastatic cancers are associated with mutations in the cell cycle and DNA repair regulatory factors; this allows the cancer cells to bypass the apoptosis mediated cell death to senescence [8,51,52]. In this work, we observed that vRAGE is an alternative repair factor to RAGE^WT^ and its robust DNA repair function thus, provides a gain of function to the cells expressing it, compared to cells expressing RAGE^WT^ only. Moreover, in addition to RAGE, other repair factors such as BRCA1, RAD51 and Mdm2 are known to express their multiple splice variants in SCLC and other cancers [53,54,55,56,57]. Thus, the switch from RAGE^WT^ to vRAGE in the progress of malignancy fits well into the known picture of alternative splicing as causative for the development of chemoresistance on several levels [47]. Cumulatively in this way a cell replaces its regulatable repair factors with the constitutively active form, which might not be under the same regulatory process as seen in their wild-type counterparts, for example, the pro-apoptotic gene Bcl-x, or the caspase-9 gene encodes for both anti and pro-apoptotic variants [58,59,60].

In summary, vRAGE fits well into this concept, due to alternative splicing, the constitutive nuclear localization of vRAGE provides an additional advantage for timely DNA repair, acting as an energetically more favorable monomer than RAGE^WT^ in the nucleus. This sustains efficient cell viability and cell properties needed for growth and metastasis. The potential of blocking vRAGE and the NHEJ pathway in SCLC anti-cancer therapy needs to be explored.

### Study Limitations

Our current study focuses on vRAGE, but there are many other variants of RAGE that have been identified. Due to the lack of a specific drug affecting alternative splicing a vRAGE generation, we could not perform animal studies in which vRAGE inhibition would be associated with sensitivity towards therapy and reduced metastasis.

## 4. Materials and Methods

Details on the plasmid used, antibodies, DNA damage treatment, immunofluorescence staining, cell culture, invasion-migration assay and information on primer sequences used are described in this section. The gene ID used in this study was Human RAGE^WT^ is NP_001193865.1 and the Human vRAGE is NM_001206936.2; NP_001193865.1.

### 4.1. Cell Culture and Transfection

Hela (WT/RAGE^−/−^) cells were grown in DMEM, whereas small cell lung carcinoma specific SHP77 cells were grown in RPMI1640 medium. Both these media were supplemented with fetal calf serum (10%), penicillin-streptomycin (1%), non-essential amino acids (1%) and L-glutamine (1%). The isolation and cultivation of RAGE^−/−^ primary fibroblasts was done as described earlier [11]. The plasmid DNA/siRNA transfection in Hela cells was performed using the direct method, whereas the suspension culture of SHP77 cells were reverse transfected. The Hela RAGE^−/−^ cells were generated by the CRISPR-Cas9 system as described earlier [61]. The gRNA seq used is given in Table S3, cloned into the pSpCas9(BB)-2A-Puro (Addgene, Watertown, MA, USA; #62988) vector backbone. The transfected cloned gRNA vector was transfected and selected on Puromycin. The generated clones were characterized, validated, sequence verified for the gene editing and then amplified for further study. In the present study we used Hela RAGE^−/−^ clone-1, details are described in figure (Figure S3a,b). Cell transfection was performed by using Turbofect transfection reagent (Thermo GmbH, Darmstadt, Darmstadt, Germany). Transfected cells were analyzed after 24–72 h of transfection, as indicated in the figure legends. The siRNA mediated depletion of RAGE was performed by using the siRAGE1 (Santacruz Biotechnology, Dallas, TX, USA; Sc36374) or siRAGE2 (Ambion AM16708, Assay ID: 110857; Thermo GmbH, Darmstadt, Germany) using the RNAi MAX siRNA transfection reagent. siRAGE2 showed better depletion than siRAGE1.

### 4.2. Cell Lysis and Immunoblotting

Total cell extracts were obtained by re-suspending them in 20 mM Tris-Cl pH 7.5, 40 mM NaCl, 2 mM MgCl_2_, 0.5% NP40, 50 U/mL Benzonase, supplemented with protease and phosphatase inhibitors and after 15 min of incubation on ice, the NaCl concentration was adjusted to 150/450 mM and then it was further incubated for 15 min. The lysates were then centrifuged at 14 K for 15 min at 4 °C. The supernatant was collected in labeled tubes and the total protein was quantified using Bradford’s reagent.

### 4.3. In Vitro Wound Healing

To test the directional migration behavior of cells, an in vitro wound closure assay was performed on 80–90% of the confluent monolayer of the indicated Hela cells in 24 well plates. A wound was introduced by a sterile pipette tip in the monolayer. Wound closure was evaluated after 0, 24, or 48 h by light microscopy.

### 4.4. Matrigel Invasion

Polycarbonate transwell permeable supports (8 μm pore size), coated with 100 μL 1:5 diluted matrigel, were incubated over-night (37 °C, under 5% CO_2_ and humidified atmosphere). Cells (5 × 10^4^, 200 μL RPMI or DMEM/1%BSA) were placed on the gels, the lower chamber containing respective medium/20%FCS. After 24 h (37 °C, 5%CO_2_), cells in the insert were removed. Matrix invasion and recovery on the lower membrane side was evaluated microscopically and photometrically after crystal-violet staining.

### 4.5. Cellular Immunofluorescence (IF)

Cells grown on poly L-lysine coated coverslips (Thermo) or glass-bottom dishes (Ibidi GmbH, Gräfelfing, Germany), treated with laser or radiomimetic drugs (Table S1) were processed differently. After drug treatment or laser-induced DNA damage cells were fixed with 4% paraformaldehyde for 15 min at 4 °C and permeabilized with 0.3% Triton X-100 in PBS for 5 min at room temperature. Samples were then blocked in 5% bovine serum albumin and immune-stained using the indicated primary antibodies and secondary antibodies (Table S2). The imaging of these stained slides was performed by using the Cell Observer, Carl Zeiss microscopy (Jena, Germany). Samples were scanned using an ×63 oil objective. Images were further processed using ImageJ (Fiji; NIH, Bethesda, MD, USA).

### 4.6. Tissue Sections Immunofluorescence

Paraffin-embedded tissue sections were de-paraffinized using a series of washes as described in [14] These prepared sections were then blocked with antibody dilution/incubation buffer (10% goat serum in TBS-0.2% Triton X100) for 45 min at room temperature. After incubation, the vRAGE antibody was diluted in the antibody dilution/incubation buffer and incubated at 4 °C for 8/10 h. Where indicated, the competitor peptide (KDGLRTREPTA) was used as specific competitor or the Flag elution peptide (Sigma, Taufkirchen, Germany; F4799) as a non-specific competitor, at 20 µg/µg of the vRAGE antibody concentration. The sections were then washed with TBS-T (10 min; three times). Fluorochrome-conjugated and species adsorbed secondary antibodies were then used to detect the signal. The negative control staining was performed simultaneously. The imaging of these stained sections was performed as described for the cellular immunofluorescence staining.

### 4.7. H&E Staining

Hematoxylin-Eosin staining of the samples was performed by using the H&Estainint kit (Abcam, Cambridge, MA, USA; Ab245880) according to manufacturer’s instructions. The stained slides were mounted, dried and then analyzed under the microscope.

### 4.8. Monoclonal Antibody Generation

The vRAGE specific rat monoclonal antibody was generated using the antigenic peptide specifically to human RAGE variant-V (vRAGE) (KDGLRTREPTA) (This sequence is located at c-terminal, close to the NLS of vRAGE) by the Monoclonal Antibody Core Facility of Helmholtz Zentrum, Neuherberg, Germany. The clones were screened, selected and propagated for large scale affinity purification. In the current study, we are used an affinity-purified RAGV9B6 clone.

### 4.9. Laser Micro-Irradiation

The cells were plated on glass-bottom dishes (IBIDI µ-wells) and pre-sensitized with 10 µM 5-bromo-2′- deoxyuridine (BrdU, Sigma-Aldrich, Taufkirchen, Germany) in phenol red-free medium (Gibco/Thermo GmbH, Darmstadt, Germany)) for 24 h at 37 °C. Micro-irradiation was performed with a FluoView1200 confocal microscope (Olympus Europa GmbH, München, Germanz) equipped with an incubator (EMBL Heidelberg, Heidelberg, Germany) heated to 37 °C. The laser settings used were the same as described previously [11].

### 4.10. Electrophoretic Mobility Shift Assay (EMSA)

For EMSA reactions the recombinant variant-5 of hRAGE or hRAGE^WT^ was mixed in a 20 μL reaction with 40–45 ng of radiolabeled probe, 0.1 mg/mL BSA and EMSA Buffer (10mM Tris-Cl pH 7.4, 50 mM KCl, 0.5 mM MgCl_2_, 0.1 mM EDTA, 5% glycerol) for 20 min at 25 °C. A 64 base pair radiolabeled probe (RAGE EM-F and RAGE EM-R) was used as a source of dsDNA. The reactions components were resolved on 6% native PAGE in 0.5 × TBE at 25 °C. The gels were dried and analyzed by exposing them to a photographic film.

### 4.11. Molecular Modelling and Docking

Amino acid sequence alignment of RAGE^WT^ and vRAGE was performed using Jalview software (Version 8.0.5; University of Dundee, Scotland, UK). A crystal structure of the extracellular region of RAGE^WT^ (VC1C2; PDB accession code: 4lp5) was used as a template structure for generating the model for vRAGE. A homology model was generated by using Modeller 9.25 [62] and the molecular docking was performed by using the web server HDOCK and HPDOCK [63,64]. Moreover, to gain the reliable comparability in docking studies, dsDNA was taken from the crystal structure of ds bound RAGE^WT^ (PDB Accession code: 4oi7). The model structure refinement was performed by using the web server PRERMD [65]. PyMOL (PyMOL molecular graphics version 2.0 Schrödinger, LLC, Cambridge, MA, USA) was used for structural analysis and generating the associated figures.

### 4.12. Graph Plotting and Statistical Analysis

All graphs were plotted using GraphPad Prism (version-7; San Diego, CA, USA). The statistical analysis was performed using the same software. Statistical difference between two groups was determined by unpaired two-tailed Student’s *t*-test or one-way ANOVA. A *p*-value < 0.05 was considered significant and different levels of significance were expressed as follows: * *p* < 0.05; ** *p* < 0.01; *** *p* < 0.001 or **** *p* < 0.0001.

## 5. Conclusions

The vRAGE modulates the DNA repair potential in SCLC cells. The expression of vRAGE, which serves as an early DNA damage response factor, along with DNA repair machinery, prominently mitigates the effects of chemotherapeutic agents; therefore, the DNA repair potential of SCLC is surprisingly high. Altogether, this study shows that constitutive nuclear localization of vRAGE and its ability to bind DNA as monomer renders vRAGE to elicit a stronger activation of DNA repair cascade, enhanced cancer cell migration and invasion than RAGE^WT^. The results of this study indicate that downregulation of the expression and/or specific molecular targeting of vRAGE in SCLC cases could be a very promising step for its timely regression of these resistant SCLC.

## Figures and Tables

**Table 1:** Anthropometric details of the study patients.

Anthropometric Variables	Control (*n* = 20)	SCLC (*n* = 80)		
Male	12	63		
Female	8	17		
	Control	SCLC Stage-I	SCLC Stage-II	SCLC Stage-III
Total	20	22	40	18
Male	12	16	29	18
Female	8	6	11	0

## Data Availability

The data presented in this study are available on request from the corresponding author.

## Supplementary Materials

The following are available online at https://www.mdpi.com/article/10.3390/cancers13112843/s1, Figure S1: A comparative analysis of the sequence and the structure of RAGE^WT^ and vRAGE, Figure S2: Recruitment of vRAGE to the site of damage in various cells, Figure S3: Sequence analysis of the RAGE^−/−^ clones generated by CRISPR-Cas9 method, Figure S4: A comparative analysis of DNA repair potential of un- or -complemented RAGE^−/−^ cells, Figure S5: Characterization of vRAGE antibody and the human SCLC material, Figure S6: siRNA mediated knockdown of RAGE in NCI-H82 cells, Figure S7: Characterization of drug resistance and metastatic potential of cells, Figure S8 Uncropped immunoblots, Table S1: Substances used for DNA damage induction, Table S2: Details of primary and secondary antibodies, Table S3: Primer sequences used in this study.
