# Supplementary material for: Elevated Expression of the RAGE Variant-V in SCLC Mitigates the Effect of Chemotherapeutic Drugs
Source: Cancers (Basel). 2021 Jun 7;13(11):2843. doi: 10.3390/cancers13112843 (PMC8201239; doi:10.3390/cancers13112843)
Supplement: Supplementary file 1 [file cancers-13-02843-s001.zip › cancers-1209166-supplementary.pdf]

cancers

Supplmentary Material: Elevated Expression of the *RAGE* Variant-*V* in SCLC Mitigates the Effect of Chemotherapeutic Drugs

Bindhu K. Madhavan, Zhe Han, Bishal Singh, Nico Bordt, Serap Kaymak, Obul Reddy Bandapalli, Lars Kihm, Khurrum Shahzad, Berend Isermann, Stephan Herzig, Peter Nawroth and Varun Kumar

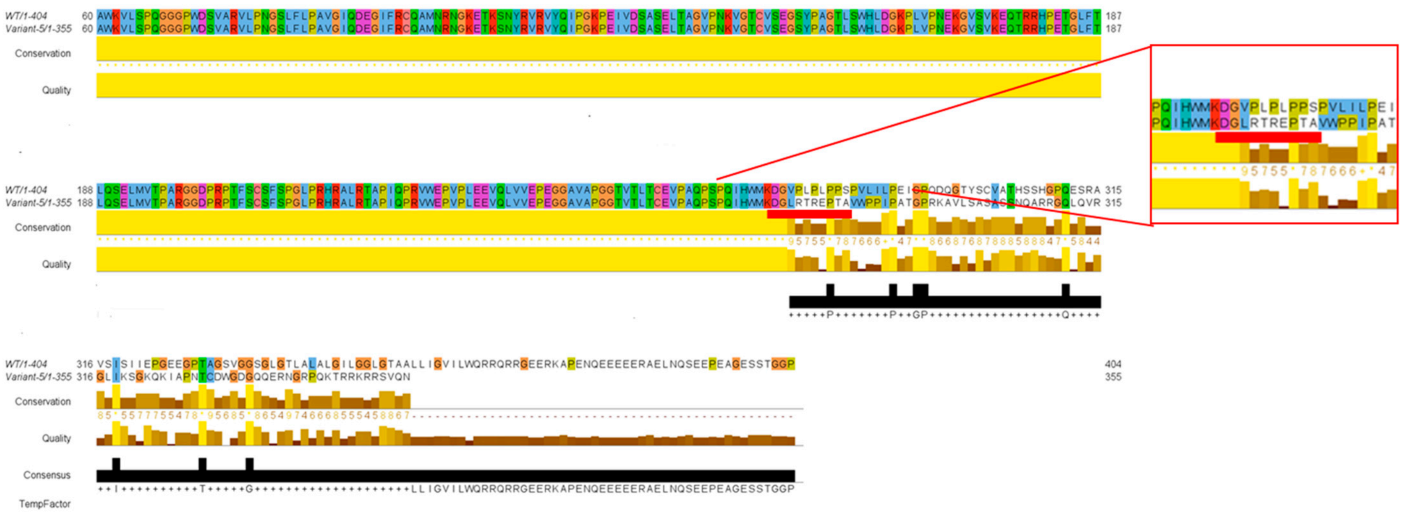

(**a**)

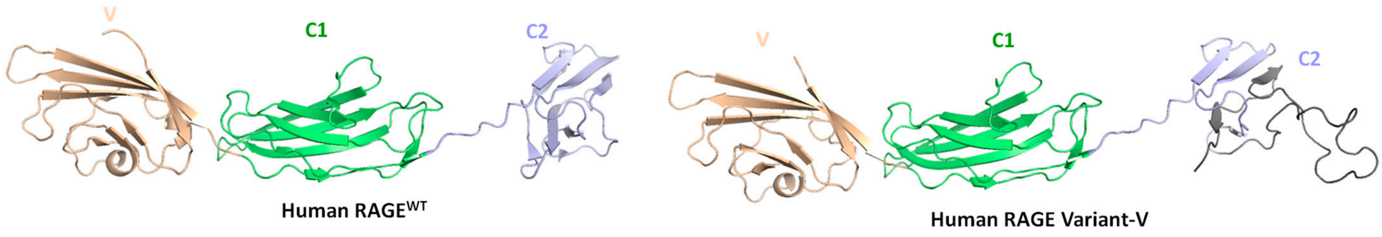

(**b**)

Figure S1. A comparative analysis of the sequence and the structure of RAGE^{WT} and vRAGE**.** (**a**) The primary amino acid sequences of human RAGE^{WT} and human RAGE variant-V were aligned using Jalview, showing the identical region (shown in the yellow ribbon below the alignment) and the un-identical residues at the c-terminus underlined in black ribbon. The zoom window (in red box) indicates the sequence of peptide specific to vRAGE used for generating the antibody and is dissimilar between RAGE^{WT} and vRAGE. (**b**) Ribbon-diagram illustration of the human RAGE^{WT} extracellular domain (VC1C2 fragment; PDB accession code 4lp5) and a homology model of the RAGE variant-V (amino acids between 20 to 330) generated using wt RAGE (PDB accession code 4lp5) as a template. The conserved amino acids in variant-V RAGE (20 to 275) are shown in cyan color and mismatched amino acids (276 to 330) are shown in grey. The areas marked in red circles are the C2 domains of RAGE^{WT} and vRAGE which are dissimilar to each other.

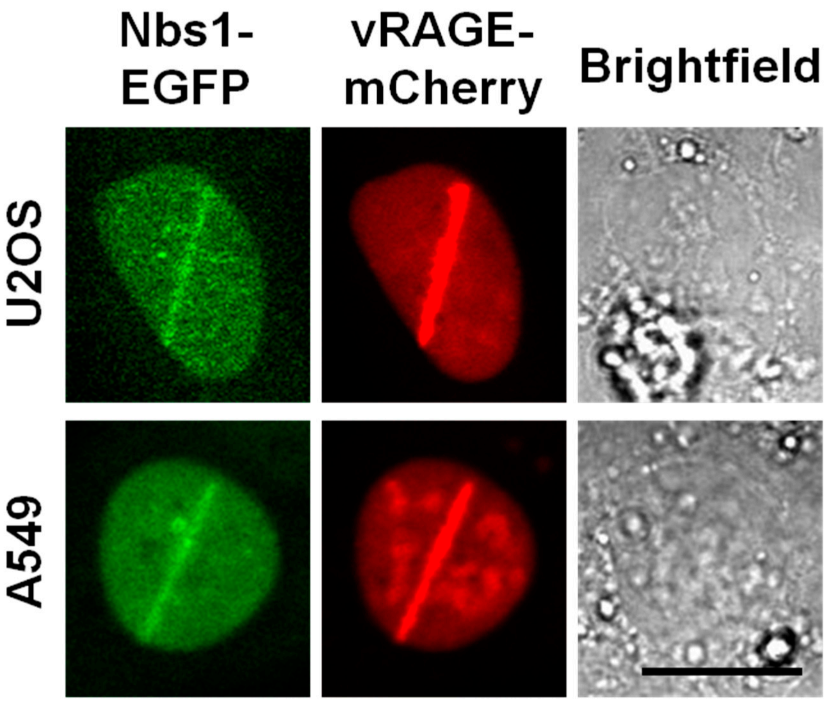

Figure S2. Recruitment of vRAGE to the site of damage in various cells. The representative still images of human Nbs1-EGFP and vRAGE-mCherry expressing U2OS (upper panel) or A549 (lower panel) cells, showing the recruitment of vRAGE at the site of damage. The Brightfield panel is presented in grey (Scale 10μm).

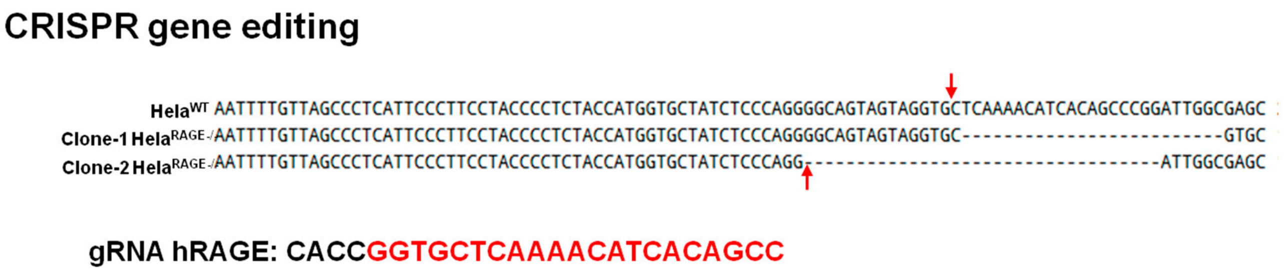

(**a**)

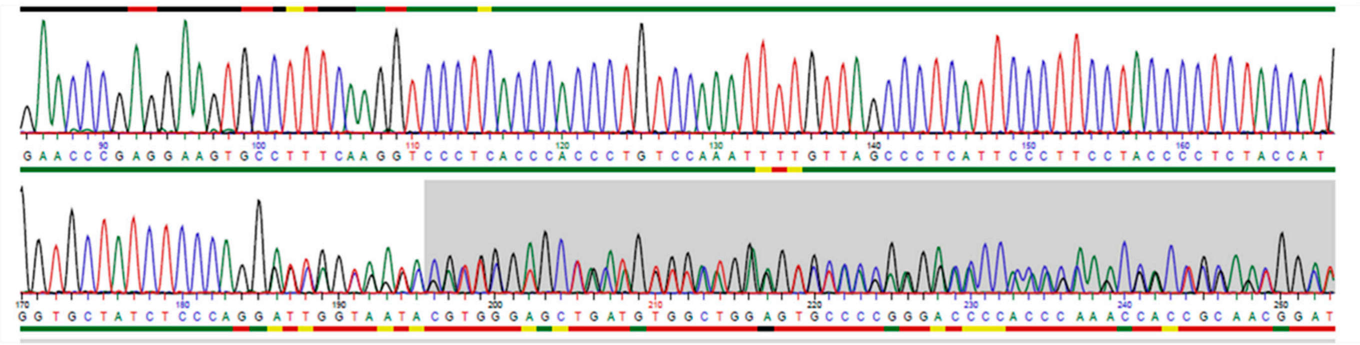

(**b**)

Figure S3. Sequence analysis of the RAGE$^{-/-}$ clones generated by CRISPR-Cas9 method. (**a**)The genomic DNA sequence analysis snapshot of CRISPR-Cas9 mediated genome-edited Hela cells showing homozygous genome editing at a RAGE gene locus. The arrow shows the site of nucleolytic cleavage. The sequence in red represents the gRNA sequence. (**b**) Indel analysis of the genomic sequence of the DNA isolated from CRISPR edited Hela cells showing homozygous editing of these cells.

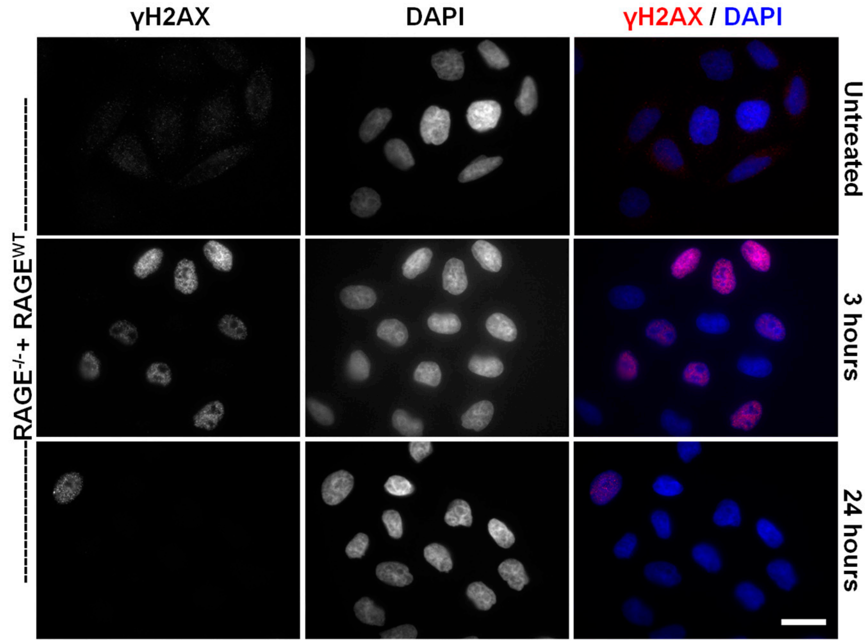
γH2AX
DAPI
γH2AX / DAPI
RAGE-/-+ RAGEWT
Untreated
3 hours
24 hours

(a)

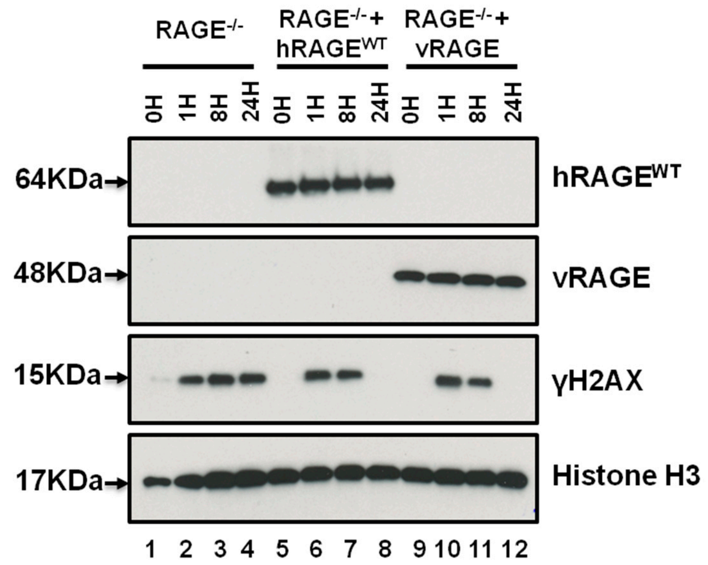
RAGE-/-
RAGE-/-+ hRAGEWT
RAGE-/-+ vRAGE
0H 1H 8H 24H 0H 1H 8H 24H 0H 1H 8H 24H
64KDa
48KDa
15KDa
17KDa
hRAGEWT
vRAGE
γH2AX
Histone H3
1 2 3 4 5 6 7 8 9 10 11 12

(b)

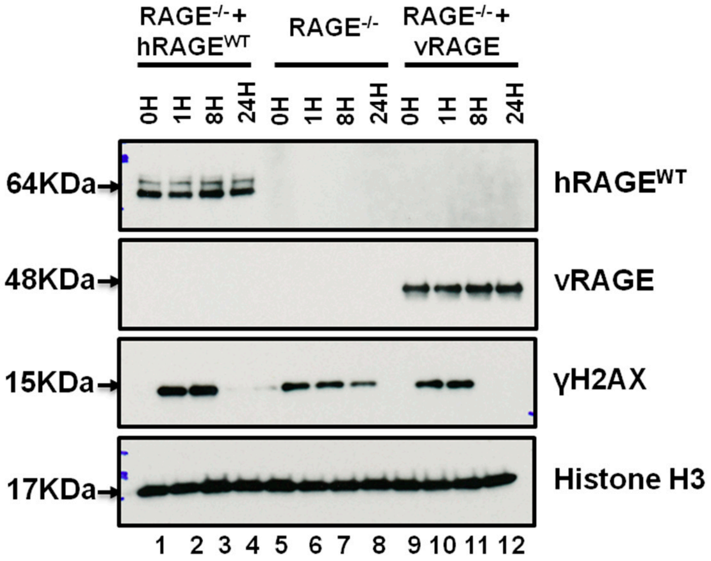
RAGE-/-+ hRAGEWT
RAGE-/-
RAGE-/-+ vRAGE
0H 1H 8H 24H 0H 1H 8H 24H 0H 1H 8H 24H
64KDa
48KDa
15KDa
17KDa
hRAGEWT
vRAGE
γH2AX
Histone H3
1 2 3 4 5 6 7 8 9 10 11 12

(c)

Figure S4. A comparative analysis of DNA repair potential of un- or -complemented RAGE$^{-/-}$ cells. (**a**) Immunofluorescence staining of Hela$^{-/-}$ cells complemented with RAGE^{WT}, followed by treatment with etoposide. Cells were analyzed for the DNA damage marker γH2AX at the indicated time points. This experimental data complement s the main figure (Figure 2a). (Scale 10μm). (**b**) Representative immunoblots from the lysates of control or RAGE depleted SCLC SHP77 cells, treated with etoposide (2.5μM for 1 hour) and probed for the DNA damage marker γH2AX at the indicated post-damage intervals. The expression/depletion of vRAGE was confirmed by using the vRAGE specific antibody (see methods). Histone H3 was used as a loading control. (**c**) Representative immunoblots from the lysates of un-complemented (RAGE$^{-/-}$) or vRAGE (RAGE$^{-/-}$ + vRAGE) or RAGE^{WT} (RAGE$^{-/-}$ + RAGE^{WT}) complemented RAGE$^{-/-}$ primary fibroblasts, treated with etoposide (2.5μM for 1 hour) or bleomycin (30μg/ml for 1 hour) and probed for the DNA damage marker γH2AX at the indicated intervals after the DNA damage. The expression of transfected vRAGE was confirmed using generated vRAGE antibody, where as the expression of RAGE^{WT} was confirmed using FLAG tag antibody. Histone H3 was used as a loading control. The primary fibroblasts were isolated from RAGE$^{+/+}$, or RAGE$^{-/-}$ mice.

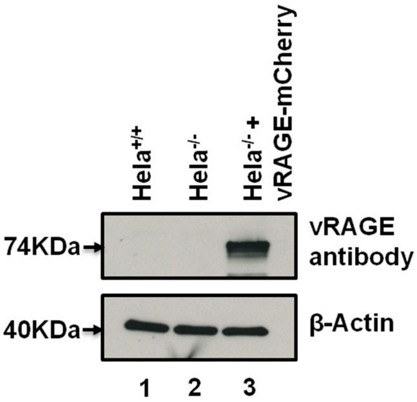

(**a**)

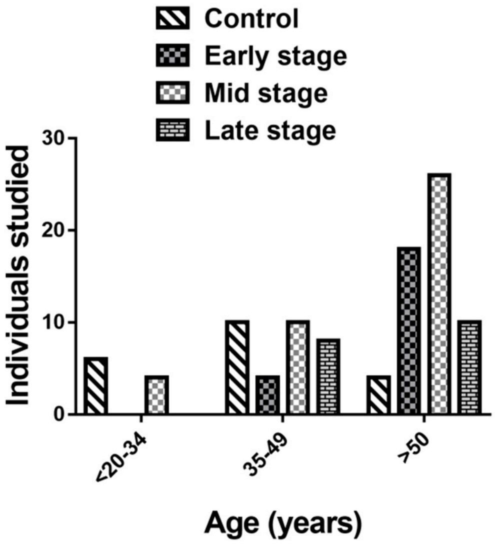

(**b**)

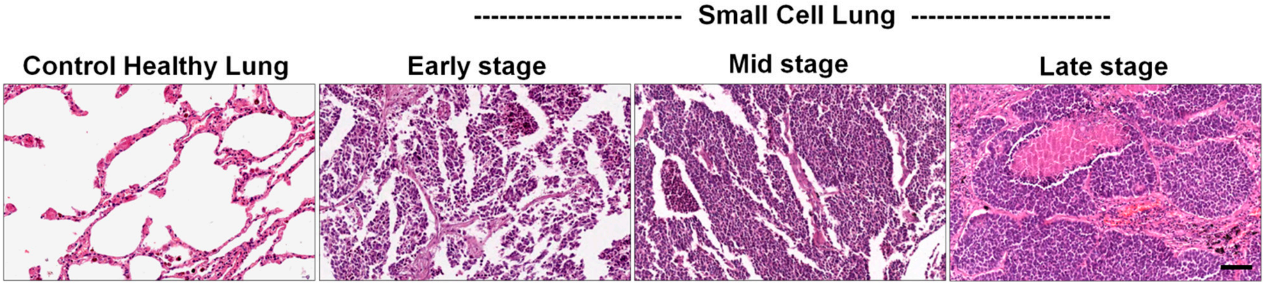

(**c**)

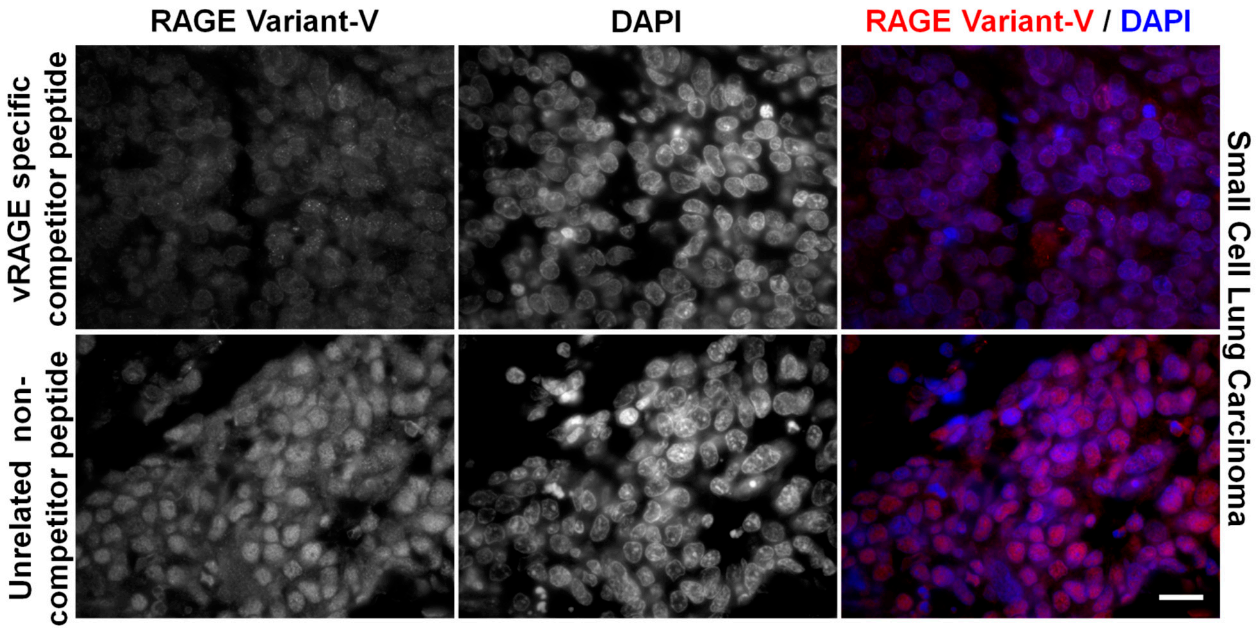

(**d**)

Figure S5. Characterization of vRAGE antibody and the human SCLC material. (**a**) Representative immunoblots from the lysates of Hela WT ($Hela^{+/+}$), $RAGE^{-/-}$ ($Hela^{-/-}$), or $Hela^{-/-}$ complemented with vRAGE ($Hela^{-/-}$ + vRAGE) probed with the human RAGE variable-V antibody. The result shows a specific signal of vRAGE in these complemented $Hela^{-/-}$ cells only. The details of this antibody generation is given in methods. (**b**) The graphical representation of the number of patients (described in table-1) at different stages of SCLC progression plotted across different age groups. (**c**) The representative H and E images of different stages of human SCLC or the control lung showing cancer progression. (**d**) The representative immunofluorescence images of human SCLC sections stained with the vRAGE antibody with the indicated competitor peptides. The red-stained areas represent the vRAGE specific signal. The DAPI (in blue) represents the nucleus (Scale 10µm).

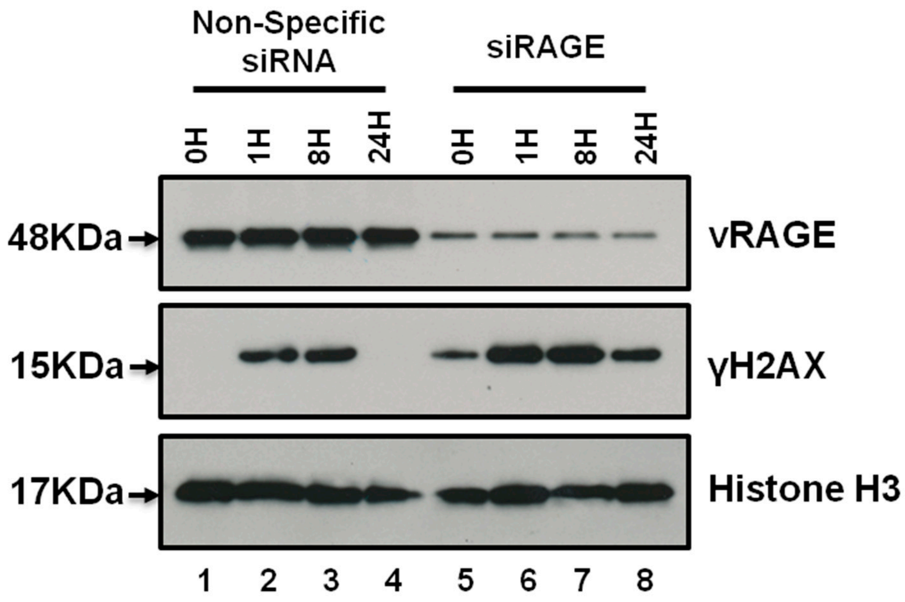

Figure S6. siRNA mediated knockdown of RAGE in NCI-H82 cells. Representative immunoblots from the lysates of control or RAGE depleted NCI-H82 cells, treated with etoposide (2.5µM for 1 hour) and probed for the DNA damage marker γH2AX at indicated post-damage intervals. The expression/depletion of vRAGE was confirmed by using the vRAGE specific antibody generated during this study. Histone H3 was used as a loading control.

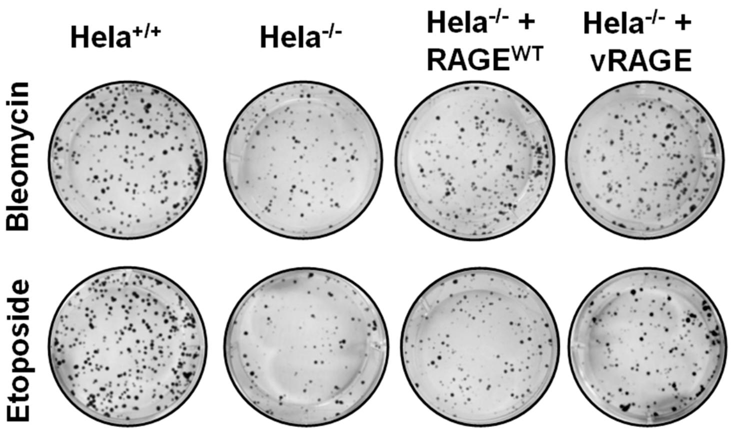
Hela+/+
Hela-/-
Hela-/- +
RAGEWT
Hela-/- +
vRAGE
Bleomycin
Etoposide

(**a**)

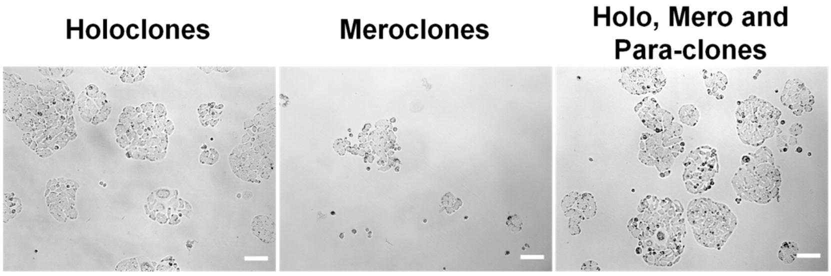
Holoclones
Meroclones
Holo, Mero and
Para-clones

(**b**)

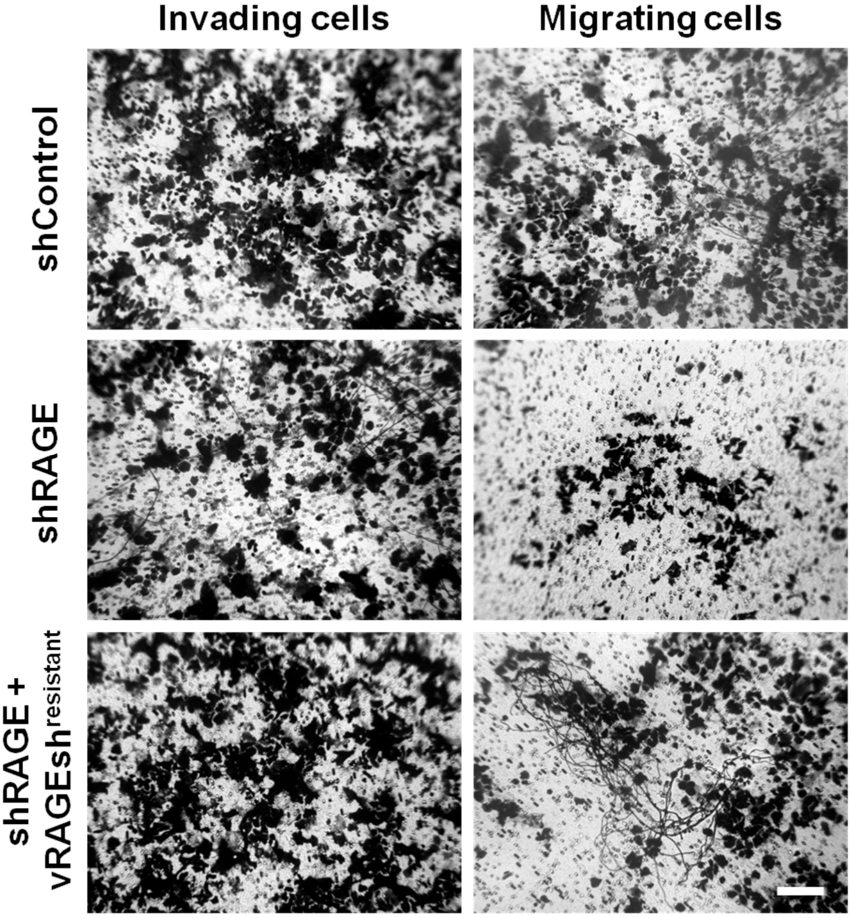
Invading cells
Migrating cells
shControl
shRAGE
shRAGE +
vRAGEshresistant

(**c**)

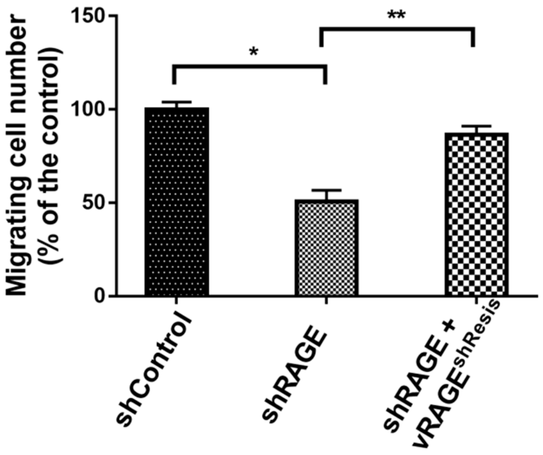

(**d**)

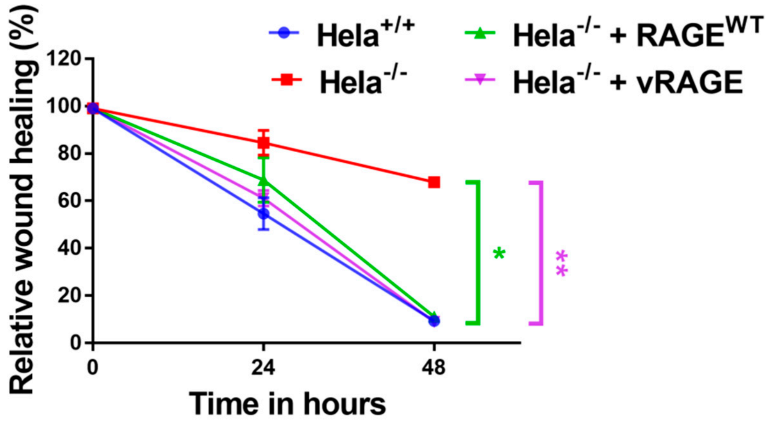

(**e**)

Figure S7. Characterization of drug resistance and metastatic potential of cells. (a)Representative images of the total colonies from the Hela$^{+/+}$, Hela$^{-/-}$, Hela$^{-/-}$ + RAGE^{WT} or Hela$^{-/-}$ + vRAGE cells survived after bleomycin (30µg/ml for 1 hour; upper panel) or etoposide (2.5µM for 1 hour; lower panel) induced DNA damage. The colonies were stained after 10 days of DNA damage recovery. (b) Representative images of colonies observed in Hela$^{-/-}$+ vRAGE cells showing an elevated frequency of Holoclone formation than para- or mero-clone (Scale 20 µm). (c) The representative images of the RAGE depleted SCLC (SHP77) cells migrated or invaded in the Transwell migration assay after complementation with or without vRAGE. The migrated cells were stained with Crystal violet (Scale 40µm). (d)The quantitative analysis of the %age of the RAGE depleted SHP77 cells migrated in the transwell assay described in figure (Supplementary figure 4c). The migrated cells were stained with Crystal violet and counted manually. (n = 3, mean ± SD, *: $p < 0.05$;**: $p < 0.01$). (**e**) The quantitative data showing the relative wound healing of the indicated cells presented in figure (Figure 6d) (n = 3, mean ± SD, *: $p < 0.05$;**: $p < 0.01$).

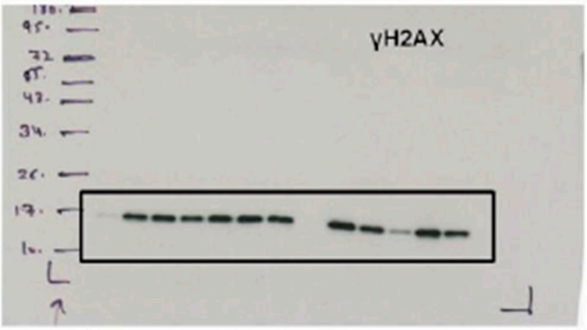
γH2AX

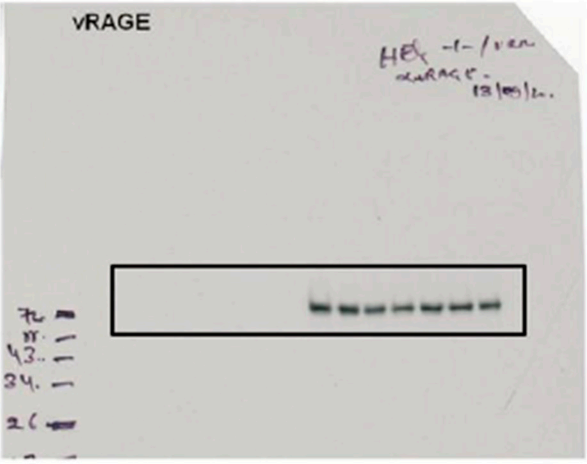
vRAGE

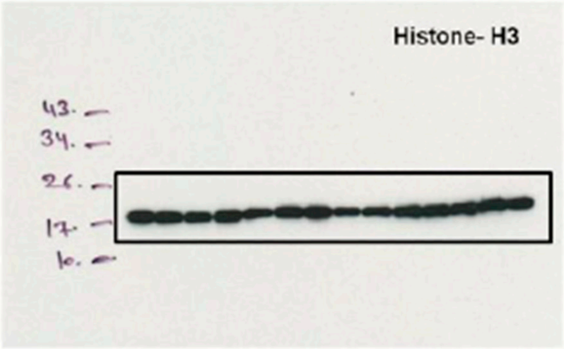
Histone- H3

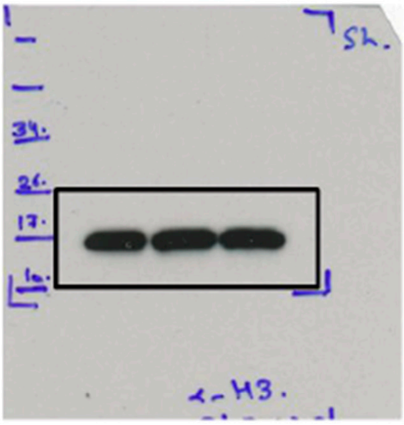
α-H3.

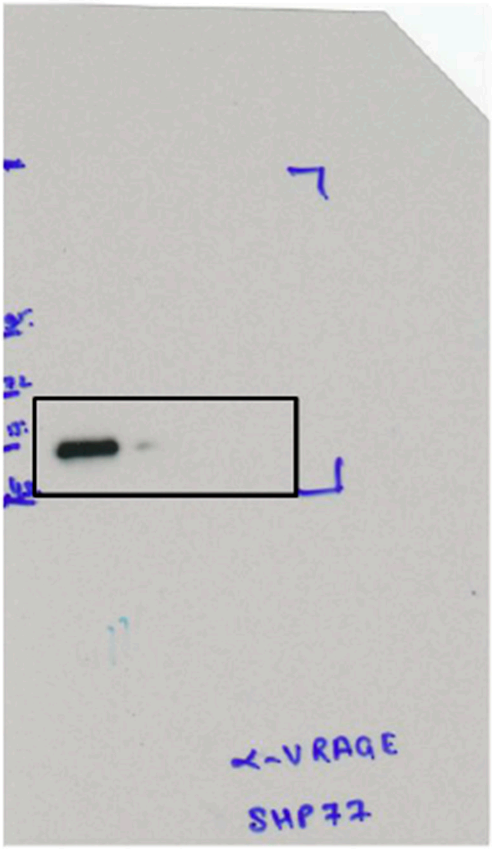
α-VRAGE
SHP77

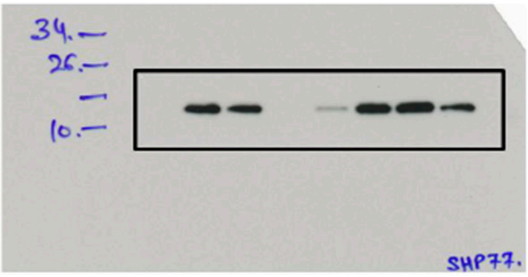
34.
26.
10.
SHP77.

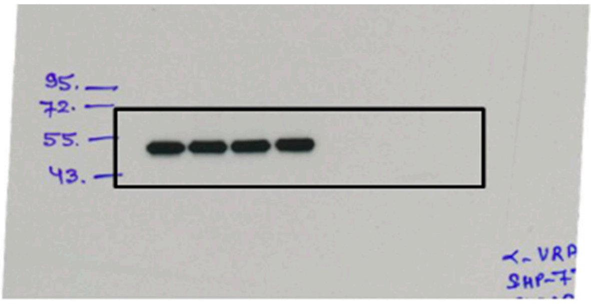
95.
72.
55.
43.

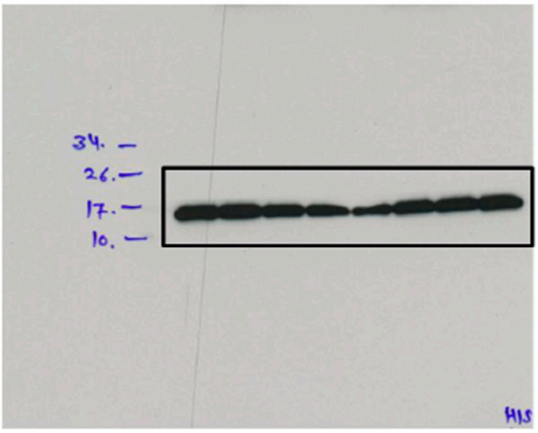
34.
26.
17.
10.
HIS

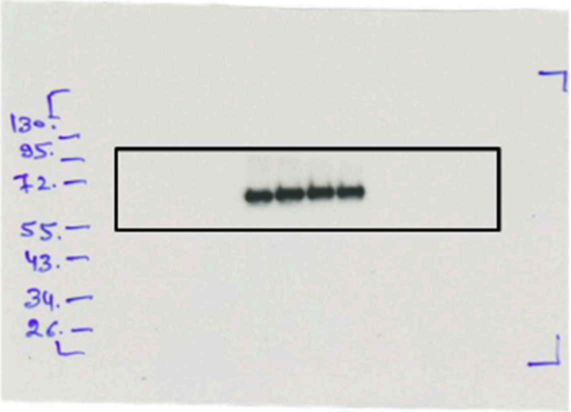
130.
95.
72.
55.
43.
34.
26.

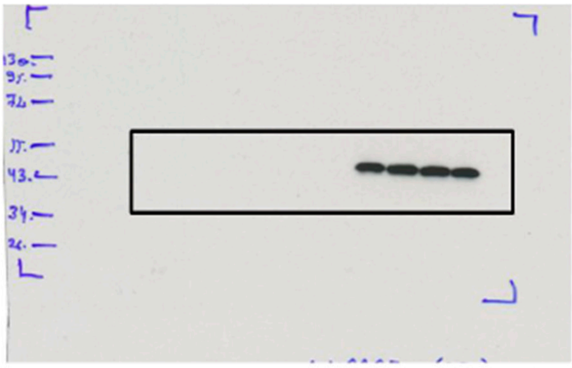
43.

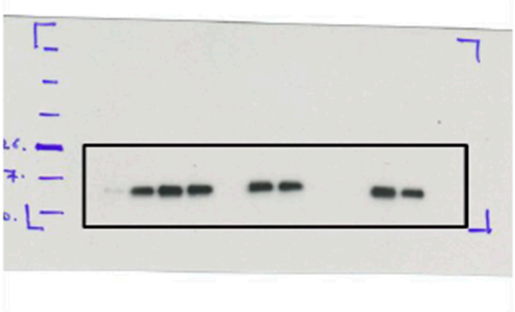

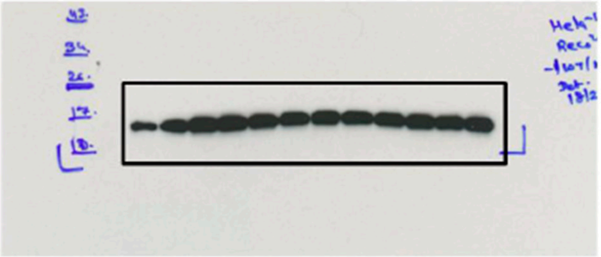
34.
26.
17.

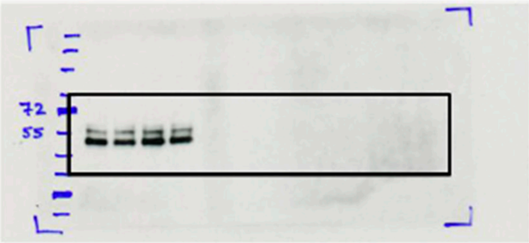
72
55

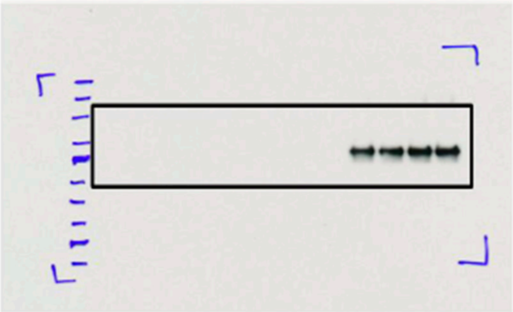

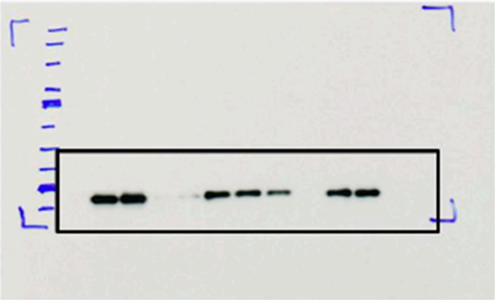

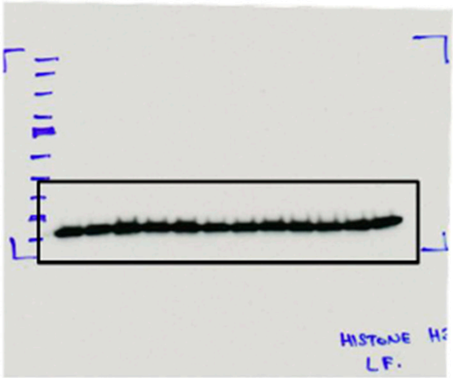
HISTONE
LF.

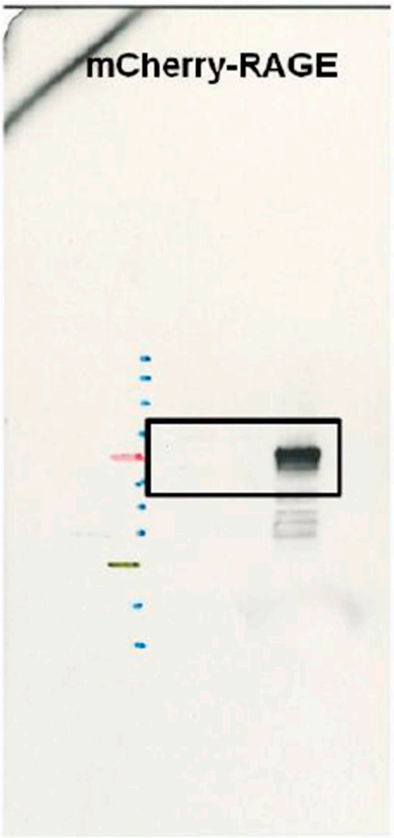
mCherry-RAGE

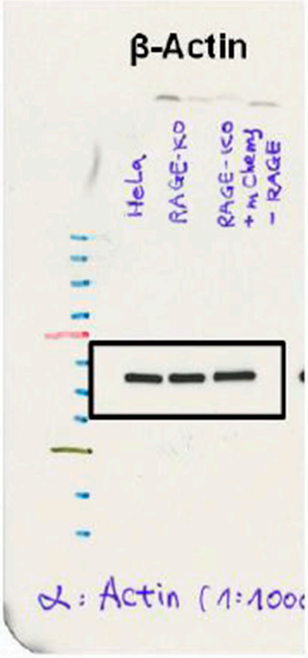
β-Actin
HeLa
RAGE-KO
RAGE-KO + mCherry - RAGE
α: Actin

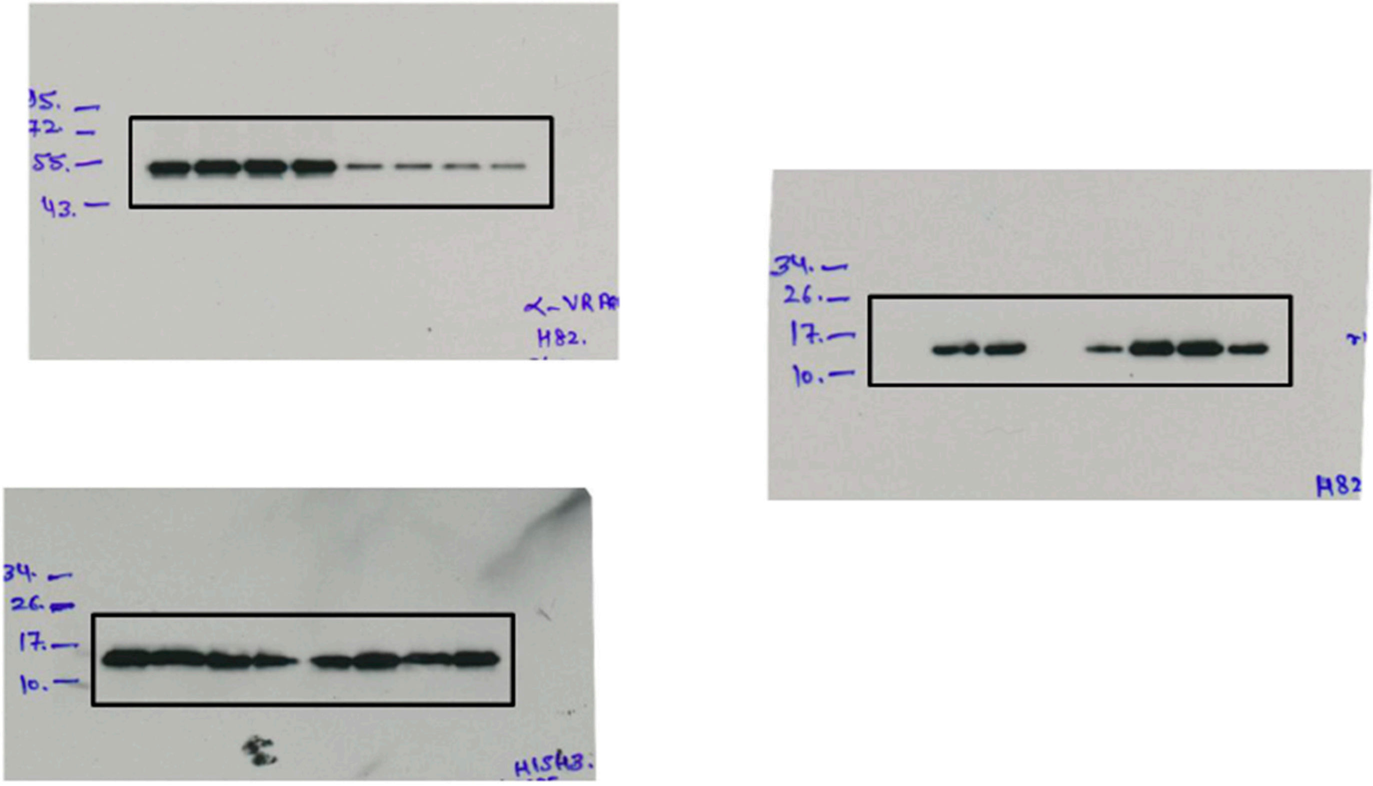

Figure S8. Uncropped immunoblots

Table S1. Substances used for DNA damage induction.

Drug	Purpose	Concentration	References
Bleomycin	DNA DSB induction	1μM (for 60mins)	2
Etoposide	DNA DSB induction	2.5μM (for 60mins)	1

Table S2. Details of primary and secondary antibodies.

Antibody	Species	Supplier	Application
Histone H3	Rabbit	4499(Cell Signaling)	IB
◎H2AX	Rabbit	9718(Cell Signaling)	IB, IF
RAGE variant-V	Rat		IB, IF
MRE11	Rabbit	4785 (Cell Signaling)	IF
**Secondary antibodies**			
Anti-Mice IgG-HRP	Goat	sc-2062 (Santa Cruz)	IB
Anti-Rabbit IgG-HRP	Goat	7074(Cell Signaling)	IB
Anti-mouse IgG-HRP	Goat	7076 (Cell Signaling)	IB
Anti Goat-Alexa-647	Donkey	Ab150139 (Abcam)	IF
Anti mice-Alexa-647	Donkey	Ab150111 (Abcam)	IF
Anti Rabbit-Alexa-555	Donkey	Ab150074 (Abcam)	IF

Anti Rat-Alexa-647	Donkey	Ab150155 (Abcam)	IF

Table S3. Primer sequences used in this study.

S.No.	Name	Sequence
1	hRAGEvariant-5 F	ATGGCAGCCGGAACAGCAGTTGGAGCCTG
2	hRAGEvariant-5 R	AGTGATGATGATGATGATGCTTGTCATCGTCATCC
3	hRAGE F	ATGGCAGCCGGAACAGCAGTTGGAGC
4	hRAGE R	AGGCCCTCCAGTACTACTCTCGCCT
5	hRAGE gRNA	CACCGGTGCTCAAAACATCACAGCC
6	RAGE EM F	GGACTCCAGGTCCAGGACCGCCGTTTTTCGCGCGCACGGCGCGGGAGGTCCA GCTGGTCCACCTCC
7	RAGE EM R	GGAGGTGGACCAGCTGGACCTCCCGCGCCGTGCGCGCGAAAAACGGCGGTCC TGGACCTGGAGTCC

Reference

1. Dobbin, M.M.; Madabhushi, R.; Pan, L.; Chen, Y.; Kim, D.; Gao, J.; Ahanonu, B.; Pao, P.C.; Qiu, Y.; Zhao, Y.; Tsai, L.H. SIRT1 collaborates with ATM and HDAC1 to maintain genomic stability in neurons. *Nat. Neurosci.* **2013,** *16*, 1008–1015.
2. Polo, S.E.; Blackford, A.N.; Chapman, J.R.; Baskcomb, L.; Gravel, S.; Rusch, A.; Thomas, A.; Blundred, R.; Smith, P.; Kzhyshkowska, J. Regulation of DNA-end resection by hnRNPU-like proteins promotes DNA double-strand break signaling and repair. *Mol. Cell.* **2012**, *45*, 505–516.
